# ClC-Kb pore mutation disrupts glycosylation and triggers distal tubular remodeling

**DOI:** 10.1172/jci.insight.175998

**Published:** 2024-11-22

**Authors:** Yogita Sharma, Robin Lo, Viktor N. Tomilin, Kotdaji Ha, Holly Deremo, Aishwarya V. Pareek, Wuxing Dong, Xiaohui Liao, Svetlana Lebedeva, Vivek Charu, Neeraja Kambham, Kerim Mutig, Oleh Pochynyuk, Vivek Bhalla

**Affiliations:** 1 Division of Nephrology, Department of Medicine, Stanford University School of Medicine, Stanford, California, USA.; 2 Department of Integrative Biology and Pharmacology, University of Texas Health Science Center at Houston, Houston, Texas, USA.; 3 Department of Physiology, UCSF, San Francisco, California, USA.; 4 Department of Pharmacology, Institute of Pharmacy, I.M. Sechenov First Moscow State Medical University, Moscow, Russia.; 5 Department of Pathology, Stanford University School of Medicine, Stanford, California, USA.; 6 Department of Translational Physiology, Charité-Universitätsmedizin Berlin, Berlin, Germany.

**Keywords:** Nephrology, Chronic kidney disease, Ion channels

## Abstract

Mutations in the *CLCNKB* gene (1p36), encoding the basolateral chloride channel ClC-Kb, cause type 3 Bartter syndrome. We identified a family with a mixed Bartter/Gitelman phenotype and early-onset kidney failure and by employing a candidate gene approach, identified what we believe is a novel homozygous mutation (*CLCNKB* c.499G>T [p.Gly167Cys]) in exon 6 of *CLCNKB* in the index patient. We then validated these results with Sanger and whole-exome sequencing. Compared with wild-type ClC-Kb, the Gly167Cys mutant conducted less current and exhibited impaired complex N-linked glycosylation in vitro. We demonstrated that loss of Gly-167, rather than gain of a mutant Cys, impairs complex glycosylation, but that surface expression remains intact. Moreover, Asn-364 was necessary for channel function and complex glycosylation. Morphologic evaluation of human kidney biopsies revealed typical basolateral localization of mutant Gly167Cys ClC-Kb in cortical distal tubular epithelia. However, we detected attenuated expression of distal sodium transport proteins, changes in abundance of distal tubule segments, and hypokalemia-associated intracellular condensates from the index patient compared with control nephrectomy specimens. The present data establish what we believe are novel regulatory mechanisms of ClC-Kb activity and demonstrate nephron remodeling in humans, caused by mutant ClC-Kb, with implications for renal electrolyte handling, blood pressure control, and kidney disease.

## Abstract 2

We report what we believe to be, a novel mutation (Gly167Cys) in the chloride channel CLC-Kb that is associated with inhibition of channel function, defective glycosylation and distal tubular remodeling.

## Graphical abstract

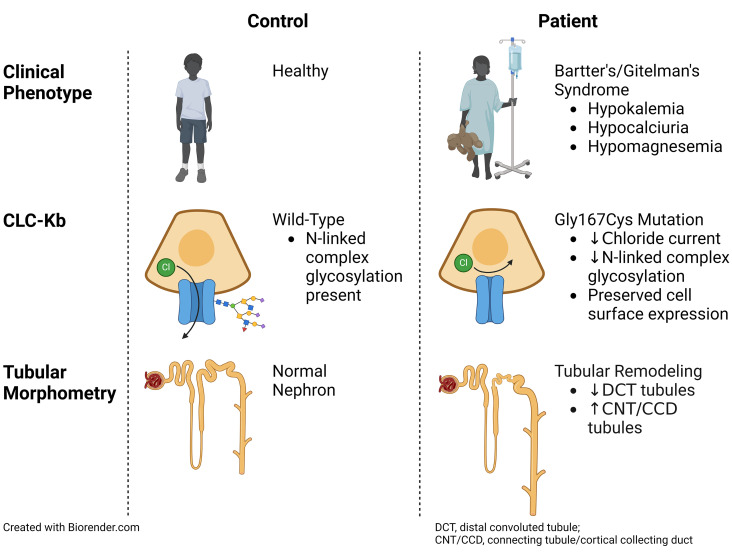

## Introduction

Bartter and Gitelman syndromes are inherited, monogenic autosomal recessive tubulopathies resulting in defects of renal electrolyte handling, with a propensity for chronic kidney disease (1). Bartter syndrome commonly demonstrates hypercalciuria with normal serum magnesium levels and secondary hyperaldosteronism with normal-to-low blood pressure, whereas patients with Gitelman syndrome exhibit hypocalciuria and hypomagnesemia (2, 3). The different biochemical profiles are the result of genetic defects that cause impaired channel activity at different nephron segments. Bartter syndrome is due to impaired function of the Na^+^-K^+^-2Cl^–^ cotransporter (NKCC2) in the thick ascending limb (TAL) of the loop of Henle, whereas Gitelman syndrome is due to impaired function of the Na^+^-Cl^–^ cotransporter (NCC) in the distal convoluted tubule (DCT) (3–6).

Five different types of Bartter syndrome have been identified due to inactivating mutations in *SLC12A1*, *KCNJ1*, *CLCNKB*, *BSND*, or *CASR*. These genes encode NKCC2, the potassium channel ROMK, the chloride channel ClC-Kb, Barttin, which is an essential β-subunit for ClC-Ka and ClC-Kb chloride channels, and the calcium-sensing receptor (CaSR), respectively (7–11). Recently, an X-linked antenatal form of Bartter syndrome has been identified that causes a transient syndrome due to a mutation in the melanoma-associated antigen D2 (*MAGED2*) gene (12).

More than 50 mutations in the *CLCNKB* gene have been reported in patients with Bartter syndrome. These mutations are scattered throughout the protein sequence, including the selectivity filter, dimer interface, C-terminal region, and Barttin binding sites. However, the most common mutations are gene deletions of *CLCNKB* (11–13).

ClC-Kb channels are expressed on the basolateral membrane of the TAL, DCT, connecting tubule (CNT), and intercalated cells of the collecting duct (CD) where they contribute to sodium/chloride reabsorption, maintenance of blood pressure, and acid-base balance (14–16). ClC-Ka channels are predominantly present on both apical and basolateral membranes of the thin ascending limb where they contribute to the generation and maintenance of the hypertonic medullary interstitium and countercurrent mechanism for urine concentration (17, 18). CLC-K1 (rat ortholog of ClC-Ka) expression is upregulated by dehydration and downregulated by the loop diuretic furosemide, whereas expression of CLC-K2 (rat ortholog of ClC-Kb) is downregulated by high-sodium diet and upregulated by furosemide (19–21). Subcellular distribution and function of ClC-K channels are tightly regulated by Barttin, an accessory subunit (22). Barttin stimulates complex N-linked glycosylation of ClC-K channels. Since complex glycosylation can be a protective mechanism against protein degradation, this might explain the high protein stability of ClC-K channels when associated with Barttin (23). Activity of human ClC-K channels is also stimulated by extracellular Ca^2+^.

The index patient presented for routine care to our pediatric nephrology service. The index patient (a 28-year-old Filipino male) and his brother presented with typical features of type 3 Bartter/Gitelman syndrome with renal electrolyte wasting and low blood pressure without nephrocalcinosis. Atypical features included progressive chronic kidney disease leading to early-onset end-stage renal disease, which is not commonly found in either Bartter or Gitelman syndrome, or the mixed phenotype (2). We characterized this family’s mutation using PCR-based sequencing and validated these findings by Sanger and whole-exome sequencing. We further performed immunohistochemical, electrophysiological, and biochemical assays to better understand the role of this mutation.

## Results

### Clinical diagnosis.

Here we report a patient with a mixed Bartter/Gitelman type 3 phenotype. The patient presented in late childhood first with fatigue, then cramps, and was found to have hypokalemia (often below 3 mEq/L, reference range 3.5 to 5.0 mEq/L), hypocalciuria (urine calcium/creatinine ratio, reference range >0.2 mg/g), and hypomagnesemia (reference range 1.7 to 2.2 mg/dL). The clinical phenotype of the patient is summarized in Table 1.

**Figure 1:**
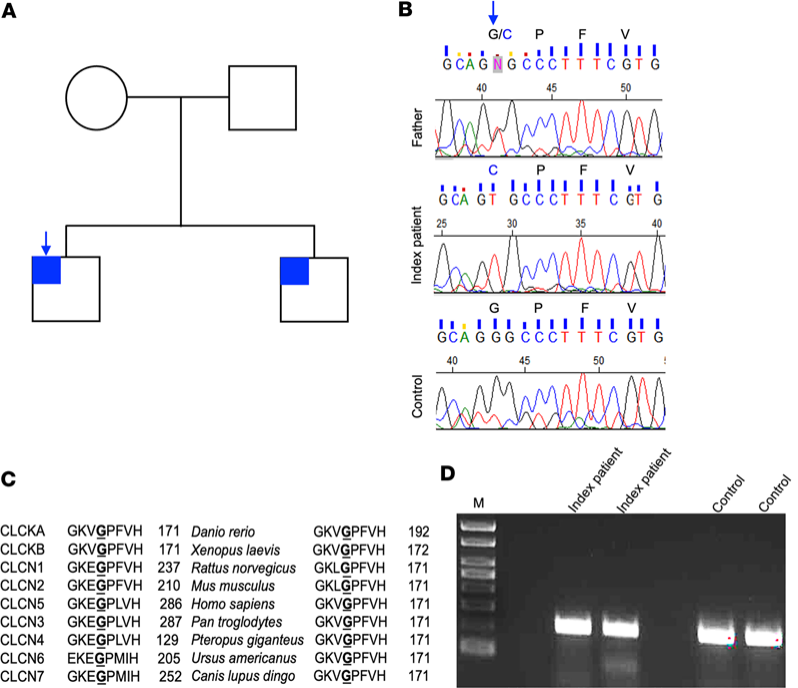
The Gly167Cys ClC-Kb mutation is inherited in an autosomal recessive pattern and disrupts a conserved glycine along the main pore of the channel. (**A**) Pedigree showing the index patient (blue arrow) and his brother affected by the phenotype (shaded blue). (**B**) Gene sequencing of *CLCNKB* exon 6 shows a homozygous G499T point mutation. (**C**) Multiple sequence alignment of human ClC family proteins and ClC-Kb across the species shows the conservation of glycine at position 167 (**G**). (**D**) RT-PCR of cDNA from peripheral blood mononuclear cells demonstrating lack of splicing from exon 4 to 6 (*n* = 1). M, marker.

The patient’s brother suffers from a similar syndrome, but no other extended family members were known to be affected (Figure 1A). Both patients developed end-stage kidney disease in adolescence. The index patient had failure to thrive and required multiple emergency room visits and hospitalizations for refractory hypokalemia despite medical therapy with sodium tablets, potassium supplements, potassium-sparing diuretics, and nonsteroidal antiinflammatory drugs. His care team performed a unilateral nephrectomy in an attempt to diminish potassium wasting. The patient had mild chronic kidney disease that then rapidly progressed to end-stage kidney disease, and the patient was initiated on hemodialysis. Both affected patients continued to maintain urine output, and both required potassium and magnesium supplementation despite the need for thrice weekly hemodialysis. The index patient also received an explant of the remaining kidney at the time of kidney transplantation.

Kidney tissue from the index patient and sonography from both the siblings revealed no nephrocalcinosis, a putative common cause of chronic kidney disease in patients with Bartter/Gitelman syndrome (2). These brothers do not suffer from sensorineural hearing loss, a feature of some patients with type 4 Bartter syndrome (e.g., with mutations in the common chloride channel cofactor Barttin, or digenic mutations in ClC-Ka and ClC-Kb) (24).

### Genetic diagnosis.

While not yet confirmed by genetics, the parents deny consanguinity and are from different islands in the Philippines. We initially pursued a candidate gene approach based on the mixed Bartter/Gitelman presentation. Based on PCR amplification and Sanger sequencing of each of the 20 exons in the *CLCNKB* gene in our index patient, we found what we believe to be a novel mutation (ntG499T) in exon 6 of *CLCNKB* that encodes a glycine to cysteine in-frame substitution. Consistent with a Mendelian form of inheritance, we validated that both brothers are homozygous for this mutation in exon 6, and the parents are heterozygous (Figure 1B). Whole-exome sequencing did not reveal any pathogenic or likely pathogenic mutations in other known Bartter and Gitelman genes (or other genes causing monogenic kidney diseases). The mutation identified (*CLCNKB* c.499G>T [p.Gly167Cys]) was absent from the genome aggregation database (gnomAD), the Human Gene Mutation Database (HGMD), and ClinVar.

The codon for Gly-167, nt 499–501 (GGC), is located on a core structural element of the ClC channel pore in the evolutionarily conserved sequence motif, GKXGPXXH (25, 26). Multiple sequence alignment shows that the glycine and this overall motif are also highly conserved within ClC family orthologs (Figure 1C). Mutations at the first nucleotide of an exon can alter splicing (27). The reference nucleotide (nt G499) is at a conserved splice acceptor site (28). To distinguish between a splicing defect and a nonsynonymous mutation due to the single nucleotide mutation, we analyzed mRNA from peripheral blood mononuclear cells. A PCR product of amplified mRNA showed the absence of splicing of exon 6 in the index patient, similar to control individuals with no known phenotype (Figure 1D). Thus, this single nucleotide mutation likely signifies a Gly167Cys mutation.

### Functional characterization of mutant ClC-Kb Gly167Cys channels.

**Figure 2:**
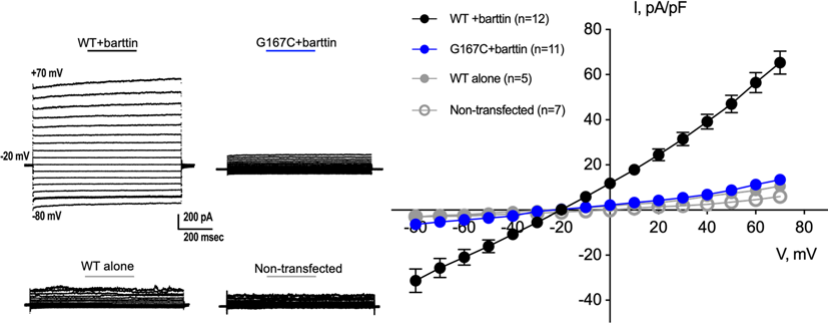
Gly167Cys mutant does not transduce significant chloride current. Electrophysiological recordings of CHO cells expressing wild-type (WT) and mutant (G167C) ClC-Kb shows loss of current in the G167C mutant even in the presence of a critical cofactor, Barttin. *P < 0.05 vs. WT + Barttin by 1-way ANOVA (*n* = 12, *n* = 11, *n* = 5, and *n* = 7 cells for WT + Barttin, G167C + Barttin, WT alone, and nontransfected, respectively).

To characterize the functional relevance of the mutation, we generated mutant ClC-Kb (Gly167Cys) and expressed wild-type or mutant ClC-Kb in CHO cells. Electrophysiological recordings at an applied voltage of –60 mV showed diminished chloride current in the Gly167Cys mutant compared with wild-type (–4.31 ± 1.00 pA/pF [*n* = 11 cells] vs. –20.86 ± 3.49 pA/pF [*n* = 12 cells], *P* < 0.05). We observed a lack of current with wild-type ClC-Kb channels in the absence of Barttin, similar to untransfected controls (Figure 2).

### Gly167Cys ClC-Kb channels lack complex N-linked glycosylation.

**Figure 3:**
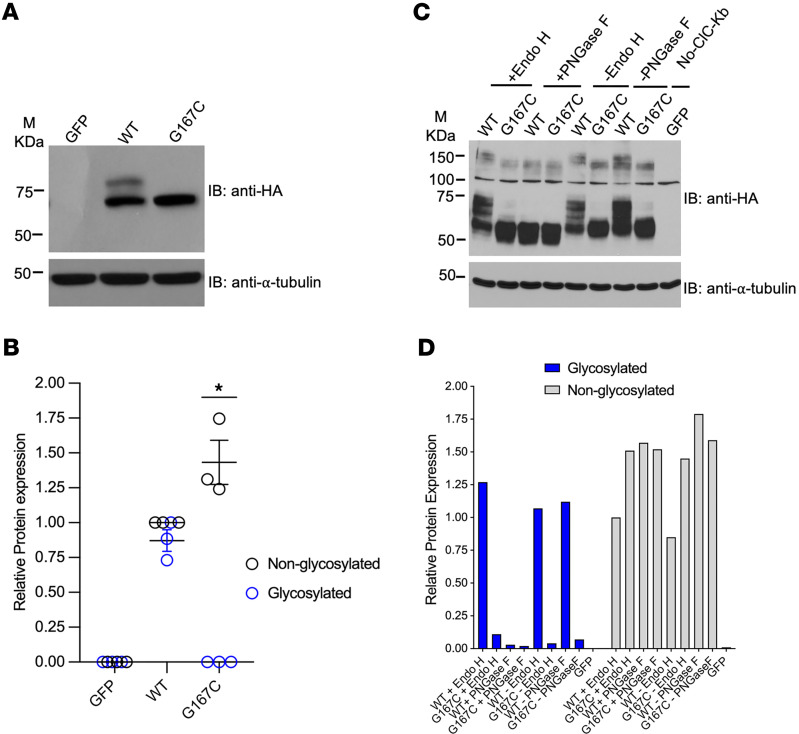
Gly167Cys mutant diminishes complex N-linked glycosylation. (**A**) Western blot of N-terminally HA-tagged wild-type (WT) and mutant ClC-Kb (G167C) probed using anti-HA and anti–α-tubulin antibodies. (**B**) Quantitative analysis of protein expression (blue, glycosylated; black, nonglycosylated) of WT vs. mutant ClC-Kb (G167C). Data are mean ± SEM of *n* = 3 experiments. *P < 0.05 for glycosylated band vs. WT; ^#^*P* < 0.05 for nonglycosylated band vs. WT by 1-way ANOVA. (**C**) Western blot of Endo H– and PNGase F–treated N-terminally HA-tagged WT and mutant ClC-Kb (G167C) probed using anti-HA antibody. (**D**) Qualitative analysis of protein expression after digestion with glycosidases (blue, glycosylated; gray, nonglycosylated) of WT vs. mutant ClC-Kb (G167C). *P < 0.05 for glycosylated band vs. WT; ^#^*P* < 0.05 for nonglycosylated band vs. WT by 1-way ANOVA. M, marker.

ClC-Kb electrophoretic mobility produces 2 bands (24). By expression studies, Western blot analysis showed the lack of an upper band in mutant ClC-Kb (0.86 ± 0.03 AU for wild-type vs. 0.01 ± 0.01 AU for Gly167Cys, *P* < 0.05) (Figure 3, A and B). We observed a lack of this upper band with Gly167Cys in CHO and HEK293T cells (see below). We demonstrated that the upper band of wild-type ClC-Kb is sensitive to PNGase F, but not Endo H treatment, suggesting that the upper band represents complex N-linked glycosylation of the wild-type channel. In contrast, we detected minimal complex N-linked glycosylation of mutant ClC-Kb (Figure 3, C and D).

### Asn-364 glycosylation is critical for ClC-Kb function.

**Figure 4:**
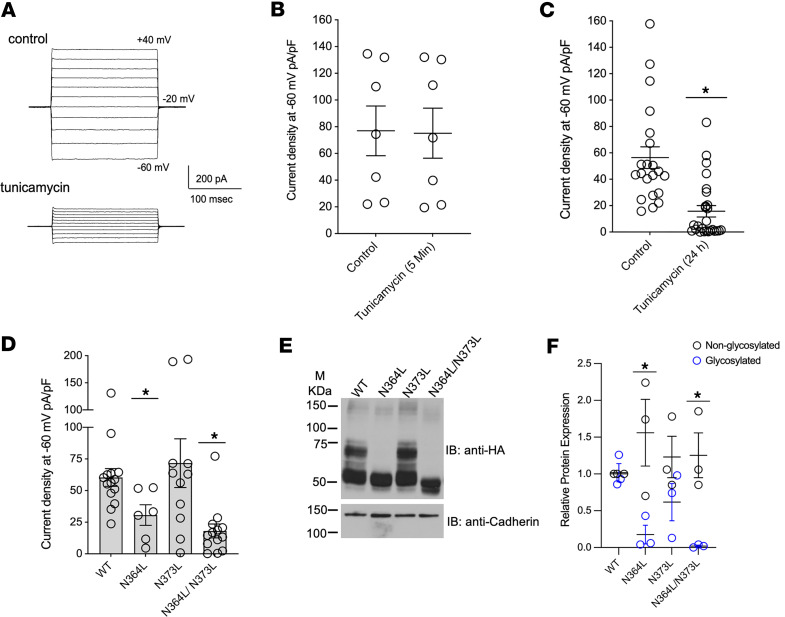
Asn-364 glycosylation is critical for ClC-Kb function. (**A**–**C**) Voltage-clamp experiment shows a reduced conductance in the presence of N-linked glycosylation inhibitor, tunicamycin, compared with vehicle-treated CHO cells (control). Data are mean ± SEM of *n* = 7–26 samples per condition. *P < 0.05 vs. control by unpaired Student’s *t* test. (**D**) Quantification of electrophysiological recordings from CHO cells expressing wild-type (WT) or potential glycosylation-site mutants of ClC-Kb (N364L, N373L, N364L/N373L). Data are mean ± SEM of *n* = 6–14 samples per condition. *P < 0.05 vs. WT by 1-way ANOVA. (**E**) Western blot of transfected WT and glycosylation mutants of ClC-Kb in HEK293T cells (probed with anti-HA and anti-cadherin antibodies). (**F**) Quantitative analysis of protein expression (glycosylated and nonglycosylated) of WT vs. potential glycosylation-site mutants (blue, glycosylated; black, nonglycosylated). Data are mean ± SEM of *n* = 3 independent experiments. *P < 0.05 vs. glycosylated band for WT; ^#^*P* < 0.05 for nonglycosylated band vs. WT by 1-way ANOVA. M, marker.

To assess whether glycosylation is necessary for ClC-Kb function, we measured chloride currents in the presence or absence of the N-glycosylation inhibitor tunicamycin. Acute application of tunicamycin for 5 minutes had no measurable effect on Cl^–^ conductance, whereas pretreatment for 24 hours led to significant reduction in ClC-Kb currents (Figure 4, A–C). This suggests that the inhibitory effect of tunicamycin may be caused by decreased glycosylation of ClC-Kb and not due to a direct, toxic effect on the channel. In the rat ortholog of ClC-Kb, Jenisch et al. eliminated the upper migratory band of ClC-Kb with a dual mutation of 2 candidate extracellular N-glycosylation sites (29). Thus, we examined mutations of these 2 extracellular N-glycosylation sites, individually and together, in human ClC-Kb (Asn-to-Leu), and recorded chloride currents. Electrophysiological recordings at an applied voltage of –60 mV showed significantly diminished chloride current with the Asn364Leu (30.68 ± 8.08 pA/pF [*n* = 6 cells] vs. 71.85 ± 17.53 pA/pF [*n* = 11 cells], *P* < 0.05), but not the Asn373Leu mutant (Figure 4D). We confirmed that the Asn364Leu site was necessary and sufficient to reduce ClC-Kb glycosylation (1.02 ± 0.12 AU for wild-type vs. 0.18 ± 0.12 AU for Asn364Leu, *P* < 0.05) (Figure 4, E and F), whereas glycosylation of Asn373Leu was similar to wild-type. These data indicate that Asn-364 is the primary site of glycosylation and is required for maximum chloride flux.

### Comparison of Gly167Cys to other ClC-Kb mutations.

**Figure 5:**
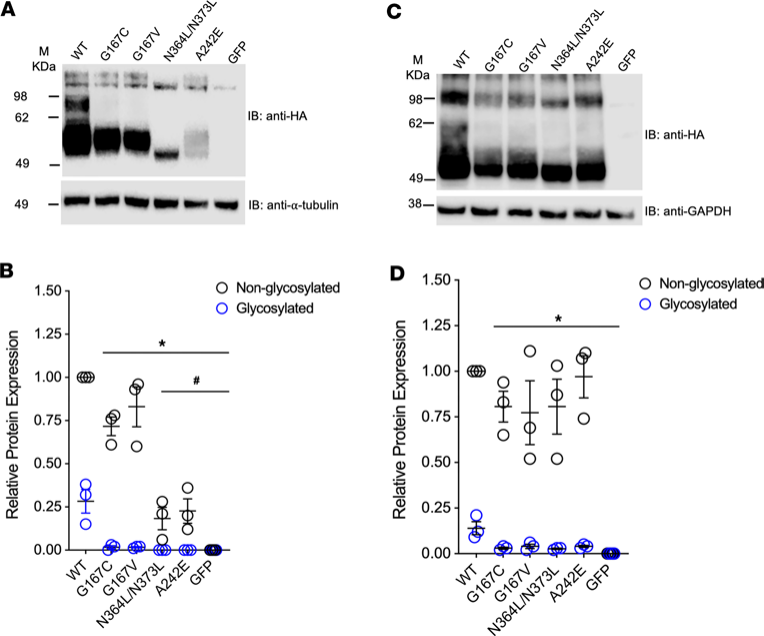
Loss of Gly-167 diminishes glycosylation. Western blot of N-terminally HA-tagged wild-type (WT) and ClC-Kb mutants (G167C, G167V, N364L/N373L, A242E) from CHO (**A**) or HEK293T cells (**C**) probed using anti-HA antibodies. Quantitative analysis of protein expression (blue, glycosylated; black, nonglycosylated) of WT vs. ClC-Kb mutants in CHO (**B**) and HEK293T cells (**D**). Data are mean ± SEM of *n* = 3 experiments. *P < 0.05 vs. glycosylated band for WT; ^#^*P* < 0.05 nonglycosylated band vs. WT by 1-way ANOVA. M, marker.

To determine whether diminished glycosylation was due to lack of a glycine or gain of a cysteine at aa 167, we further quantified glycosylation of other human ClC-Kb mutations (30). Among these, Gly167Val, similarly to Gly167Cys, also exhibited significantly diminished glycosylation in CHO cells (0.28 ± 0.07 AU for wild-type vs. 0.02 ± 0.01 AU for Gly167Cys vs. 0.02 ± 0.01 AU for Gly167Val, *P* < 0.05) (Figure 5, A and B). For comparison, mutations that lacked either N-linked glycosylation site (Asn364Leu/Asn373Leu) or Ala242Glu, a known pathogenic Bartter syndrome type 3 mutation (31), also demonstrated diminished glycosylation compared with wild-type ClC-Kb. However, these latter 2 mutants also had significantly diminished expression of nonglycosylated ClC-Kb compared with wild-type. We detected a similar pattern of results for glycosylation in HEK293T cells, although the relative amount of glycosylated versus nonglycosylated ClC-Kb varied compared with CHO cells, indicating subtle differences between these 2 cell lines (Figure 5, C and D).

### Surface expression of Gly167Cys ClC-Kb in vitro and in vivo.

**Figure 6:**
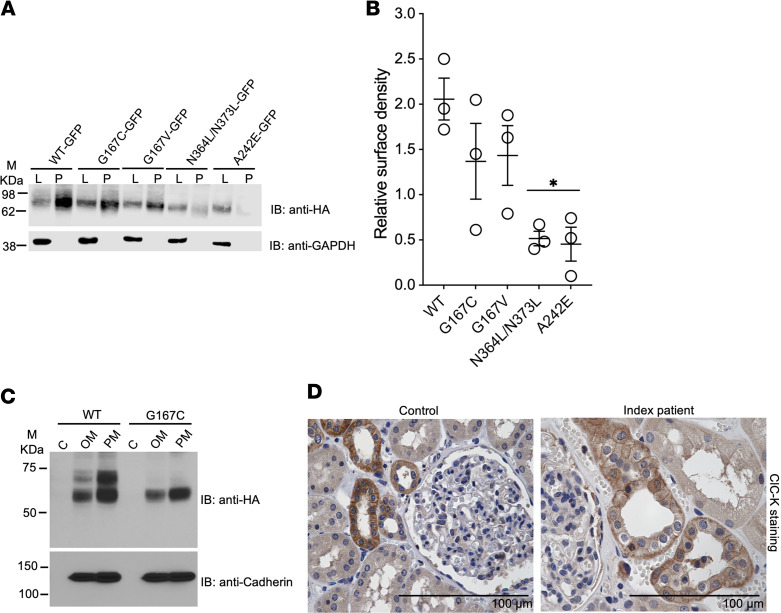
Gly167Cys mutant ClC-Kb is expressed at the surface. (**A**) Surface biotinylation and (**B**) quantification of the surface expression of wild-type (WT) and ClC-Kb mutants (G167C, G167V, N364L/N373L, A242E) using Western blotting. The surface expression of WT and ClC-Kb mutants indicated by precipitate (P) was normalized to total protein in lysates (L). Data are mean ± SEM of *n* = 3 independent experiments. *P < 0.05 vs. WT by 1-way ANOVA. (**C**) Western blot of WT and mutant (G167C) ClC-Kb in cytosolic (C), organelle membrane (OM), and plasma membrane (PM) fractions (probed using anti-HA antibody) from HEK293T cells (*n* = 1). (**D**) Staining for ClC-Kb using ClC-K antibody from kidney cortex of an age- and sex-matched control and the index patient (*n* = 1). Scale bars: 100 μm. M, marker.

Next, we assessed whether the diminished ClC-Kb–mediated current may be caused by impaired N-linked glycosylation, which may serve as a critical sorting signal for surface expression (e.g., from protein misfolding). We found similar expression of wild-type and mutant ClC-Kb at the surface of transfected HEK293T cells using surface biotinylation (Figure 6, A and B) and subcellular fractionation (Figure 6C). To evaluate the surface expression of mutant ClC-Kb in its native environment, we qualitatively assessed surface expression in the kidney tissue. In a control sample, using an anti–ClC-K antibody showed expected staining of basolateral membranes and cytoplasmic staining of cortical thick limb and DCT cells, consistent with ClC-Kb expression (16) (Supplemental Figure 1; supplemental material available online with this article; https://doi.org/10.1172/jci.insight.175998DS1). Immunohistochemical ClC-Kb labeling of kidney tissue from the index patient showed similar basolateral staining (Figure 6D).

### The Gly167Cys mutation is associated with distal tubular remodeling.

Histologic analysis of the kidney of the index patient compared with controls demonstrated enlarged glomeruli with juxtaglomerular hyperplasia (Supplemental Figure 2). We observed occasional areas of mild mesangial sclerosis and hypercellularity. The extent of tubular atrophy and interstitial fibrosis was mild. Patchy but confluent areas of distal tubules, characterized by (a) smaller and paler epithelial cells compared with proximal tubules and (b) by lack of visible brush borders, suggest distal tubule hyperplasia. Analysis of the medulla demonstrated hyperplasia of interstitial cells with periodic acid–Schiff^+^ granules. We also observed that blood vessels were largely well preserved.

**Figure 7:**
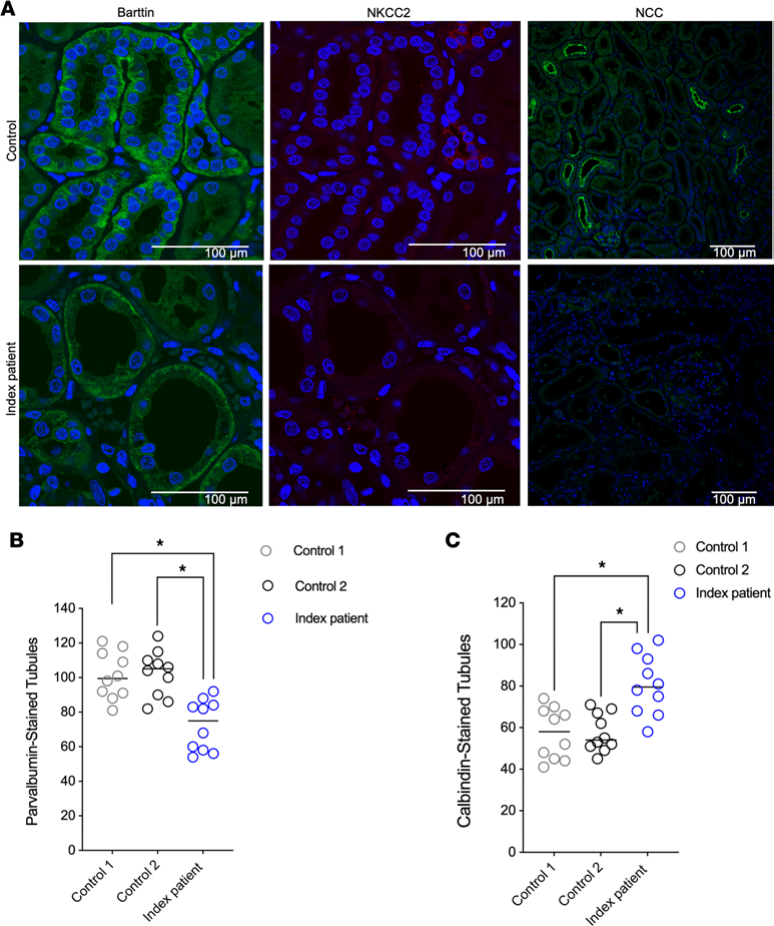
Gly167Cys mutation associates with diminished transporter expression and distal tubular remodeling. (**A**) Representative images from kidney cortex, stained with anti-Barttin, anti-NKCC2, or anti-NCC, as indicated (*n* = 1). Comparison of controls (black and gray) and index patient (blue) tubules. Decrease in the number of parvalbumin^+^ tubules (**B**) and increase in number of calbindin^+^ tubules in the index patient (**C**). *P < 0.05 vs. control kidney tissues by 1-way ANOVA (*n* = 10 tubular sections per group). Scale bars: 100 μm.

**Figure 8:**
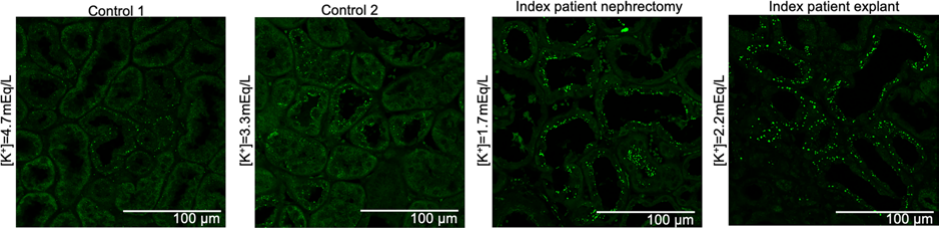
Gly167Cys mutation associates with increased condensates. Representative images from controls versus samples from index patient labeled for SPAK (green signal). Plasma potassium concentrations at the time of tissue harvest are indicated (*n* = 1). Scale bars: 100 μm.

Labeling of Barttin, NKCC2, and NCC demonstrated lower staining intensity in kidney tissue from the index patient compared with control samples (Figure 7A). Similarly, by tubular morphometry, we revealed diminished DCT mass in the kidney of the index patient compared with controls (5.3% ± 0.35% vs. 13.75% ± 2.05%) (Supplemental Table 5). We also observed a decrease in parvalbumin^+^ tubules (72.5 ± 4.6 vs. 101.9 ± 4.2 tubules, *P* < 0.05) and an increase in calbindin^+^ tubules (80.5 ± 4.5 vs. 57.3 ± 3.4 tubules, *P* < 0.05) in the index patient compared with age- and sex-matched controls (Figure 7, B and C). These data are consistent with atrophy of the DCT and compensatory hypertrophy of the downstream CNT/cortical collecting duct (CCD). Labeling of SPAK, a kinase downstream of lysine-deficient protein kinase 1 (WNK1) and WNK4 and upstream of NCC (32–34), demonstrated increased intensity and abundance of punctuated condensates resembling hypokalemia-related WNK^+^ bodies in the index patient compared with control samples (Figure 8) (35). One of our controls had mild hypokalemia, although the index patient had more severe hypokalemia at the time of nephrectomy and subsequent explant.

## Discussion

Chronic kidney disease is observed in Bartter and Gitelman syndrome patients (2). With type 3 Bartter syndrome, nephrocalcinosis is absent, as in our affected family. Thus, other possible etiologies include, but are not limited to, ischemic nephropathy from chronic volume depletion, use of nonsteroidal antiinflammatory drugs, and hypokalemic nephropathy (36, 37).

The glycine residue mutated in our family is conserved across several species from *Danio rerio* to *Homo sapiens* and is also conserved in all the human ClC family proteins (Figure 1C). Based on different characteristics, a cysteine substitution for glycine is likely highly detrimental. Cysteine is highly reactive and can form disulfide bridges, both within the molecule and with other molecules because the site of the mutation is extracellular. This Gly167Cys mutation is predicted to be pathogenic based on the MutPred2 score (0.921). This conserved glycine (Glu167) in a ClC ortholog resulted in a switch in ion selectivity (38). A valine substitution at the same glycine residue (Gly167Val) of the ClC-Kb channel has been identified in children with Bartter syndrome and leads to a reduction in chloride current compared with wild-type. These data provide complementary evidence for the functional importance of a Gly-167 mutation in ClC-Kb.

Abnormal folding due to the presence of cysteine is unlikely to be the primary mechanism for diminished chloride currents and for the salt-losing tubulopathy of affected family members. Gly167Cys had similar surface expression relative to wild-type ClC-Kb in vitro and in vivo. These data also suggest that the absence of glycine rather than the presence of cysteine may be responsible for diminished channel function. The electrophysiology and surface expression data with glycosylation-deficient mutant channels in cultured cells and human kidney is consistent with a role for Asn-364 glycosylation in channel activity rather than trafficking. The Asn-364/Asn-373 mutant channel had lower surface expression and a different migration pattern than the nonglycosylated bands of the other ClC-Kb constructs that we tested. Thus, we speculate that perhaps core glycosylation occurs at Asn-373, and that this form of N-linked glycosylation, not diminished in Gly167Cys channels, is required for surface expression, but not channel activity. Absent a role in trafficking, the glycosylation of a plasma membrane protein (e.g., at Asn-364) could alternatively affect its stability and/or function. Glycosylation is not required for cell surface expression of all channels. For example, N-linked glycosylation of Kv1.1 K^+^ channels affects the gating function, but is not required for cell surface expression (39, 40). Taken together, our results show that the mutation in our affected family may alter the glycosylation of the channel, which could then alter channel function.

We considered that Gly167Cys channels have diminished glycosylation, and therefore diminished function, due to a conformational change that indirectly alters N-linked glycosylation sites. However, the glycosylation defect of Gly167Cys channels would not be predicted from the tertiary structure, as Gly-167 is distant (~34 Å) from a consensus motif (N-X-S/T) for N-linked glycosylation (Supplemental Figure 3). As mutations in Barttin also lead to diminished ClC-Kb glycosylation in vitro (23), we tested whether Gly167Cys channels fail to be glycosylated due to diminished interaction with Barttin; however, we were unable to demonstrate direct interaction of wild-type ClC-Kb and Barttin in vitro as a comparator. Additionally, we cannot rule out that Gly167Cys channels disrupt function due to, e.g., blockade of the chloride pore or direct effects on channel gating. Gly-167 is located in the F helix N-terminus, a region known for its predominant role in the chloride pathway (25, 26). A mutation at Gly-167 may affect the backbone of the F helix that could alter the position of Val-166 or Tyr-520 that are part of the extra ion-binding site within the pore (Supplemental Figure 3) (26). Another interpretation of the data is that Gly167Cys blocks the pore, reduces chloride flux in the biosynthetic pathway, and that could negatively regulate glycosylation in the Golgi apparatus. Irrespective of the mechanism of channel dysfunction in Gly167Cys channels, glycosylation is necessary for maximal ClC-Kb function. Our data are the first to our knowledge to show mutations within ClC-Kb that are necessary for glycosylation and channel function despite intact surface expression. Thus, this study pursuant to the discovery of a previously unreported Gly167Cys mutation reveals what we believe are previously underappreciated mechanisms for the regulation of human ClC-Kb and expands the set of mutations in ClC-Kb related to Bartter/Gitelman syndrome. Furthermore, reconstitution of glycosylation side chains could augment ClC-Kb function.

Diminished sensitivity to furosemide and thiazide diuretics and consequent ENaC-dependent hypokalemia and metabolic alkalosis in mice lacking ClC-Kb (*Clck2*) (41) mimic the phenotype seen in patients with Bartter syndrome type 3 (11). Based primarily on preclinical data, changes in sodium transport within a particular segment may correlate with tubular epithelial cell plasticity. For example, with inhibition of NCC, NCC-expressing cells in the DCT show apoptosis or atrophy (41, 42), whereas increased activation of NCC increases the mass of DCTs (33, 43). The presumed loss of function of ClC-Kb in the index patient reveals morphometric data consistent with diminished transporter expression in the TAL and DCT, atrophy of DCTs, and compensatory hyperplasia of the downstream CNT and CCD. These data indicate that the Gly167Cys mutation associates with distal tubular remodeling in the index patient and are mechanistically consistent with diminished sodium chloride reabsorption in ClC-Kb–expressing TALs and DCTs and enhanced transporter function in downstream calbindin^+^ late DCTs/CNTs. One limitation of this study is the small number of control participants, as kidney biopsies or nephrectomy are not indicated in healthy individuals. Nonetheless, the present demonstration of tubular remodeling represents the first human evidence to our knowledge of both direct and indirect structural tubular adaptations to changes in the relative work load of various nephron segments, thus corroborating the remodeling hypothesis arising from data obtained in rodents.

The expression profile of distal Na^+^/Cl^–^ transport proteins observed in the index patient provides specific insights into distal nephron biology in humans, as compared with rodents. In contrast with *Clck2*-knockout mice showing significantly diminished furosemide responsiveness, but remnant NKCC2 presence in some TALs (41), the Gly167Cys ClC-Kb mutation identified in the index patient led to a virtual absence of NKCC2 expression. One explanation for this discrepancy may be that in humans, ClC-Kb is the primary chloride channel in TALs, whereas mice may have alternate basolateral chloride channels in a subset of NKCC2^+^ cells.

Enhanced intracellular accumulation of SPAK in WNK^+^ bodies is consistent with the response of the distal nephron to hypokalemia in rodents (32, 35, 44). Preclinical and clinical evidence suggest that basolateral heteromeric Kir4.1 channels respond to low extracellular potassium via depolarization, and this triggers activation of basolateral chloride channels such as ClC-Kb to lower intracellular chloride. This low intracellular chloride, in turn, activates WNK4 by autophosphorylation and subsequently SPAK and NCC in DCTs (34). WNKs rapidly assemble these condensates by functioning as crowding sensors for multimolecular assembly (44) (e.g., in response to hyperosmolarity and cell shrinkage). These condensates are utilized by DCT cells to activate WNK→SPAK→NCC in response to hypokalemia. Considering the atrophy of the DCT and mutant ClC-Kb in the index patient, the enhanced WNK^+^ bodies that we observed may be attributable to the late DCT or CNT, although double-labeling for the respective segment-specific markers would be needed for confirmation. Unlike the early DCT, the late DCT and CNT may utilize basolateral chloride exit pathways other than ClC-Kb.

### Conclusions.

We describe a previously unreported Gly167Cys mutation in human ClC-Kb that presented as a salt-losing tubulopathy without nephrocalcinosis, but with early onset of end-stage kidney disease in 2 siblings. This mutation near the permeation pore of the channel significantly reduces channel activity, whereas its surface expression is preserved despite impaired complex N-linked glycosylation. The present results suggest that intact glycosylation of ClC-Kb is required for its full activity and plays a critical role in distal sodium and chloride reabsorption. This study also uncovers the structural and functional plasticity of the human nephron with reduced DCT mass and compensatory enlargement of downstream nephron segments.

## Methods

### Sex as a biological variable.

In this study, male participants served as controls in order to match the index patient and his affected sibling was male. Although the study focused on males, both males and females may develop type 3 Bartter syndrome, and thus, we anticipate that the insights are applicable to both sexes.

### Genetic sequencing of CLCNKB.

In addition to publicly available genetic data, our local control individual for genotyping was an unrelated race/ethnicity-matched, Filipino man of similar age with no evidence of kidney disease and a normal electrolyte composition. We extracted genomic DNA using the Gentra Puregene Blood Kit (Qiagen) from peripheral blood mononuclear cells of the index patient, family members, and healthy young male volunteers, including an unrelated race/ethnicity-matched, Filipino man of similar age with no evidence of kidney disease and normal serum electrolytes. We performed PCR to amplify each of the 20 exons of the *CLCNKB* gene using primers (Supplemental Table 1). We performed PCR similarly to Yu et al. (45) and products were sequenced by Elim Biopharmaceuticals using an ABI 3730xl DNA Sequencer. We also utilized a commercial vendor to verify the sequence in all members of the nuclear family of the index patient.

For whole-exome sequencing of the index patient, we extracted genomic DNA from peripheral blood mononuclear cells, and used the Agilent SureSelectXT2 Homo Sapiens All Exon V6 kit to target the exonic regions of the patient’s genome. We then sequenced these targeted regions using the Illumina sequencing system with 150-bp paired-end reads. We mapped the DNA sequence to, and analyzed in comparison with, the published human genome build (UCSC hg38 reference sequence). The targeted coding exons and splice junctions of the known protein-coding genes were assessed for the average depth of coverage and data quality threshold values. Our mean depth of coverage was 101.5×, and the quality threshold was 97.4%. Using the Burrows-Wheeler Aligner (BWA; https://bio-bwa.sourceforge.net/), variant calls were generated followed by GATK analysis. We then annotated variants with ANNOVAR (https://annovar.openbioinformatics.org/en/latest/ Accessed August 29, 2020.), RefSeq (https://www.ncbi.nlm.nih.gov/refseq/ Accessed August 29, 2020.), and Genecode (https://www.gencodegenes.org/ Accessed August 29, 2020.) databases. Only variants in selected genes known to cause monogenic forms of kidney disease were analyzed further. A full list of selected genes is presented in Supplemental Table 2. All variants were classified according to American College of Medical Genetics and Genomics (ACMG) criteria.

We also isolated mRNA from peripheral blood mononuclear cells and performed reverse transcriptase PCR (RT-PCR) to assess for exon skipping.

### Antibodies.

We utilized rabbit polyclonal antibodies for detection of specific nephron segments (Supplemental Table 3): DCT1 with anti-parvalbumin (Thermo Fisher Scientific) and CNT/CCD with anti-calbindin (Chemicon International). For ClC-Kb, we used an anti–ClC-K antibody (Alomone) that recognizes both ClC-Ka and ClC-Kb. To localize ClC-Kb, we confined analysis to cortical sections. For detection of other relevant endogenous proteins, we used anti-Barttin (gift from Thomas Jentsch, Charité-Universitätsmedizin Berlin) (22), anti-NKCC2 (Charité-Universitätsmedizin Berlin) (46), anti-NCC (gift from David Ellison, Oregon Health Sciences University, Portland, Oregon, USA) (47), and anti-SPAK antibodies (Cell Signaling Technology). For biochemical studies we utilized anti-HA High Affinity (Roche), anti–α-tubulin (Sigma-Aldrich), anti-cadherin (Sigma-Aldrich), and anti-GAPDH (Cell Signaling Technology), followed by incubation with HRP-labeled secondary antibodies goat anti–rabbit IgG–HRP (SouthernBiotech) and goat anti–mouse IgG, Human ads-HRP (SouthernBiotech).

### Histology and immunohistochemical staining.

We obtained nephrectomy samples from age- and sex-matched controls (Supplemental Table 4). We performed histologic analysis using hematoxylin and eosin and periodic acid–Schiff staining. We performed immunohistochemical staining on slides from the index patient’s kidney nephrectomy or controls (from patients after nephrectomy for unrelated causes). We deparaffinized sections in xylene and hydrated in a graded alcohol series. We then blocked sections with normal serum for 30 minutes. We next performed heat-induced antigen retrieval by microwave pretreatment in citric acid buffer (10 mM, pH 6.0) and incubated sections with anti–ClC-K, anti-Barttin, anti-NKCC2, anti-NCC, or anti-SPAK antibodies (Supplemental Table 3) at room temperature. For all sections, we further incubated with HRP-conjugated (Vector Laboratories) secondary antibody for 30 minutes, followed by DAB detection. Cells of the TAL and DCT cells of the cortex were identified by morphology.

### ClC-Kb expression and mutagenesis.

Merritt Maduke (Stanford University) provided an N-terminally HA-tagged hClC-Kb, in a pCR4-TOPO vector. We generated mutant ClC-Kb constructs using the Quickchange site-directed mutagenesis kit (Stratagene). We verified all plasmid sequences and then subcloned cDNAs into the pMO vector into the BamHI and EcoRI sites using a standard cloning protocol. For the expression of hClC-Kb, we cultured HEK293T cells and/or CHO cells (American Type Culture Collection) in DMEM, supplemented with 10% fetal bovine serum and antibiotics (1× penicillin-streptomycin). We then transiently transfected these cells with each ClC-Kb construct and accessory subunit Y98A Barttin in a 1:1 ratio using FuGENE HD transfection reagent (Promega, E2311) and following the standard protocol.

### Electrophysiology.

We analyzed whole-cell macroscopic current recordings of ClC-Kb activity expressed in CHO cells under voltage-clamp mode using the perforated-patch technique, as previously described (48). We dissolved amphotericin B to 600 ng/mL (Enzo Life Sciences) in the pipette solution containing 150 mM KCl, 2 mM MgCl_2_, and 10 mM HEPES (pH 7.35) by ultrasonication. Recording pipettes had resistances of 4–6 MΩ. We utilized a bath solution of 150 mM NaCl, 5 mM KCl, 1 mM CaCl_2_, 2 mM MgCl_2_, 5 mM glucose, and 10 mM HEPES (pH 7.35). We obtained current-voltage (I-V) relationships by monitoring channel activity at applied holding potential from –80 mV to +70 mV, with a step of 10 mV/s in the presence or absence of 5 μg/mL tunicamycin (Tocris Bioscience), an inhibitor of N-linked glycosylation, as indicated. All macroscopic currents are normalized to whole-cell capacitance, which was approximately 9 pF for CHO cells. We acquired whole-cell current data from GΩ seals with an Axopatch 200B (Molecular Devices) patch clamp amplifier interfaced via a Digidata 1440 (Molecular Devices) to a PC running the pClamp 10.4 suite of software (Molecular Devices). We low-pass filtered currents at 1 kHz with an 8-pole Bessel filter (Warner Instruments).

### Biochemical analysis of ClC-Kb.

We generated lysates from CHO and/or HEK293T cells using RIPA lysis buffer (G-Biosciences) containing protease inhibitors. For analysis of N-linked glycosylation, we digested lysates with Endo H (New England Biolabs) or PNGase F (New England Biolabs) as indicated.

### Analysis of surface expression.

To quantify surface expression of ClC-Kb, as previously described (31), we utilized C-terminally GFP-tagged constructs for WT and mutant ClC-Kb, as indicated. Briefly, we transfected cells with ice-cold PBS, labeled with 10 mM sulfo-NHS-SS-biotin (Thermo Fisher Scientific) at 4°C for 30 minutes, and quenched with 100 mM glycine/PBS before lysis. We equilibrated streptavidin agarose beads (Thermo Fisher Scientific) with 3 washes of 1% Triton X-100 TBS, loaded with 100 μg protein for 10 minutes at room temperature, washed again with 1% Triton X-100 TBS 3 times, and eluted cell surface proteins via heating at 95°C for 10 minutes in SDS sample buffer with DTT. We also performed subcellular fractionation by extraction of plasma membrane, organelle membrane, and cytosolic protein fractions from total lysate according to the manufacturer’s instructions (Plasma Membrane Protein Extraction Kit, 101 Bio).

### Western blot analysis.

We resolved membrane proteins by 10% SDS-PAGE and then transferred onto PVDF membranes for incubation with primary followed by secondary antibodies. We visual PVDF membranes by chemiluminescence and quantified band intensity using ImageJ software (NIH).

### Tubular morphometry.

Parvalbumin, a Ca^2+^-binding protein, is expressed in the initial segment of the DCT (early but not in late DCT), whereas calbindin is present in late DCTs, CNTs, and principal cells of the CCD (49, 50). We blocked slides with 10% normal donkey serum in suppressor serum before overnight incubation of primary antibodies with an Avidin/Biotin blocking kit (Vector Laboratories). To visualize nuclei, we washed samples and used Vectashield Hard Set Mounting Medium with DAPI (Vector Laboratories). For image capture, we utilized a Leica SP8 confocal microscope with 3-color sequential scanning (DAPI, Alexa Fluor 488, Alexa Fluor 565) and analyzed using Photoshop (Adobe Systems). We counted the number of parvalbumin and calbindin positively stained tubules in controls (control nephrectomy samples) and from the nephrectomy sample of the index patient.

We also quantified DCT mass by semiquantitative morphometry. Briefly, we labeled 5-μm-thick paraffin sections for NCC whose localization is restricted to the DCT. We evaluated cortical areas extending between the renal capsule and the boundary of the outer medulla. We then photographed and superimposed sections with a transparent grid with rectangular crossed lines with the distances between lines corresponding to 50 μm. For the fractional volume of DCTs, we measured the proportion of grid intersections overlying DCT segments normalized to the recorded cortical area.

### Statistics.

For statistical comparisons with normal data distribution, we used unpaired Student’s *t* test as appropriate for 2 columns and parametric 1-way ANOVA as appropriate for more than 2 columns. We designated statistical significance at a 2-sided *P* value of less than 0.05 for all biologically meaningful comparisons. For each experiment, we note the number of samples in the corresponding figure legend. We represent data per sample when indicated and include mean ± SEM.

### Study approval.

We obtained approval for this study from the Stanford University School of Medicine Institutional Review Board. We also had written consent for nephrectomy samples from healthy controls and the index patient.

### Data availability.

All data are included in the manuscript and/or supplementary materials. Additional details, data, and deidentified material may be provided by the corresponding author upon request. All raw data for this study are provided in a Supporting Data Values Excel file.

## Author contributions

AVP, WD, and VB designed the study. YS, RL, VNT, KH, HD, AVP, WD, XL, SL, VC, NK, KM, OP, and VB performed experiments and/or analyzed the data. YS, RL, KH, KM, NK, and VB generated the figures. YS, RL, HD, and VB drafted and revised the manuscript, which was seen and approved by all authors.

## Supplementary Material

Supplemental data

Unedited blot and gel images

Supporting data values

## Figures and Tables

**Table 1:**
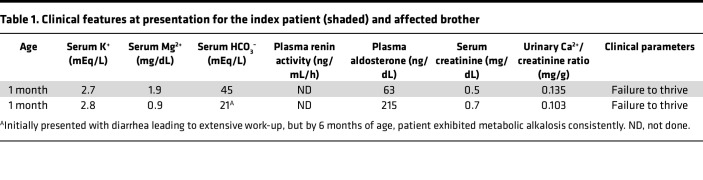
Clinical features at presentation for the index patient (shaded) and affected brother

## Version 1 10/15/2024

In-Press Preview

## Version 2 11/22/2024

Electronic publication
