# Supplementary material for: ClC-Kb pore mutation disrupts glycosylation and triggers distal tubular remodeling
Source: JCI Insight. 2024 Nov 22;9(22):e175998. doi: 10.1172/jci.insight.175998 (PMC11601903; doi:10.1172/jci.insight.175998)
Supplement: Supplemental data [file jciinsight-9-175998-s107.pdf]

SUPPLEMENTARY FIGURES and LEGENDS

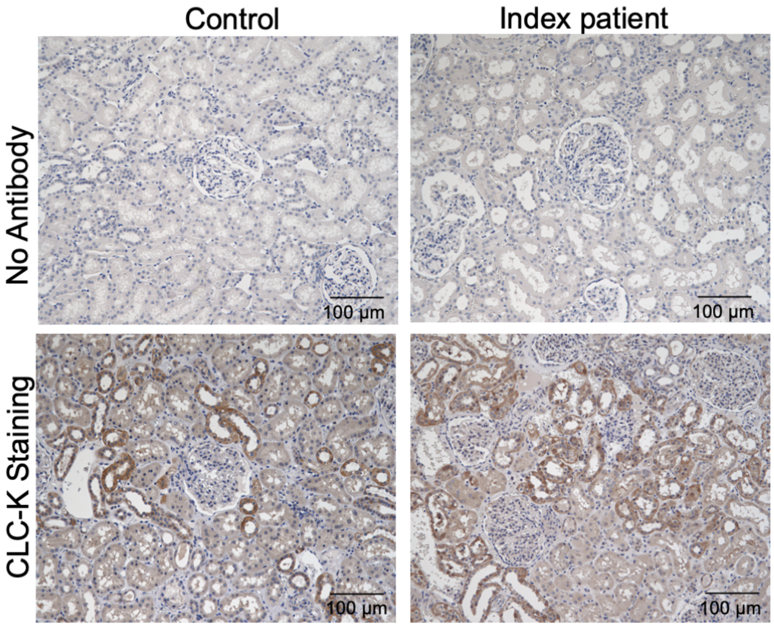

Supplementary Figure S1: Staining from the cortex of an age- and sex-matched control and the index patient with no primary vs. CLC-K antibody. Scale bar, 100 µM.

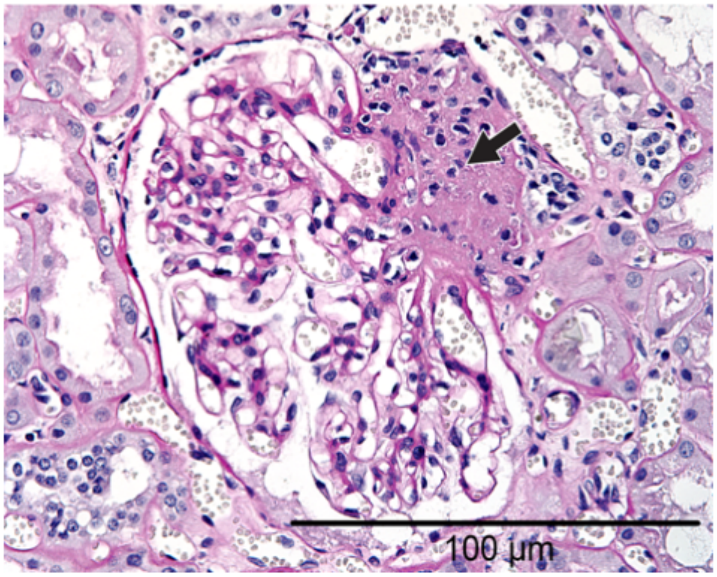

Supplementary Figure S2: Nephrectomy from the index patient shows juxtaglomerular hyperplasia. The arrow points to hyperplasia of the juxtaglomerular apparatus. Scale bar, 100 µM.

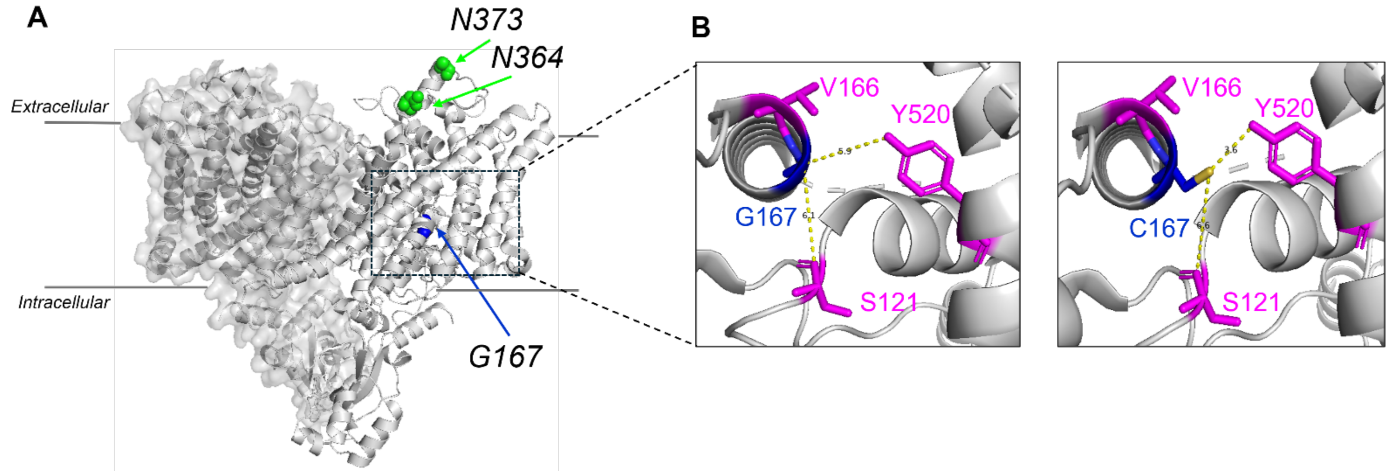

Supplementary Figure S3: The homodimeric structure of bovine ClC-K channel (5TR1). (A) Cryo-EM structure of bovine CLC-K channel shows the localization of the mutant site (G167) and putative N-glycosylation sites (N364 and N373). The mutated residue (Gly-167, blue) is located distant from the Asn-364 or Asn-373 (green) glycosylation sites. (B) G167 is localized in the ion transport pathway formed by Ser-121, Val-166, and Tyr-520 (magenta). The mutagenesis simulation shows that the sulfur of Cys-167 is localized near Tyr-520 within 3.6 Å range, suggesting impaired Cl^- binding.

SUPPLEMENTARY TABLES

Exon	Forward Primer (5'-> 3')	Reverse Primer (5'-> 3')	PCR Product (kb)
1	ACTGGAAGGGCCTAGAGGCA	GATGTCCTGAGTGGTCCTCCA	231
2	TGCCCCACCCTGTGCCGTGAC	CTTGGCCCAGAGCAGCACCTG	203
3	GAGGCTGTGGGTGCCTCCTG	AGTGGGGACTGGCGTAGCAC	200
4	CCCTCCTGGCCCTGGCCCAC	GGGTGGTTGGGATGCCCTCAC	202
5-6	CGTGTGACTCTGGGTGAGAC	GGAGGAGCTTGAGGGACCCAG	356
7	GGAGGGCCCACCTGAGATCA	GCAGGGCCAGGGTCAGGCAG	193
8,9	CGCCATCTTGGCTCCCCACTG	AGCTCGCTGAGAGGTCCCCAG	374
10	CAGCCCTAGAGCCCACCCATC	GGGCTTCCCCACTCCTGCCAC	214
11	CCTCATGTCCAGTTCCCACCT	GTCCCAGCTCTGTGCACACCTG	246
12-13	TGTCCACGCCTTGCCCAGCAG	CACGACATTGCCCACGCAGCA	439
14	CTGCCTGACTCTGCCCTTGCA	GACTCAGCCTGAGGTGGGCAC	264
15	GACTGTGGGGCCTGATGGGA	CCTACCCCCGACTTCCTCCTC	207
16-17	GAACAGTTCTTGGCTAAGTAG	CCAGAGGCCTCATGTGTCACA	454
18	GGGCACCTTCTACCCTCCAGT	GTCTTCTCAGGCATAGGTTCCC	193
19	CTACATCCCCCCGCACCTCCA	GTTATGCCAAAGAATGGAGCT	215

Supplementary Table S1: List of primers for sequencing of *CLCNKB* gene.

#	Name of gene	#	Name of gene	#	Name of gene	#	Name of gene	#	Name of gene
1	*ABCC6*	136	*COX8A*	280	*HRAS*	406	*PDSS2*	541	*SLC37A4*
2	*ABCD4*	137	*CPT1A*	281	*HSD11B2*	407	*PET100*	542	*SLC3A1*
3	*ACE*	138	*CPT2*	282	*HSD17B3*	408	*PEX1*	543	*SLC4A1*
4	*ACP5*	139	*CRB2*	283	*HSD17B4*	409	*PEX10*	544	*SLC4A4*
5	*ACTA2*	140	*CREBBP*	284	*HSD3B2*	410	*PEX11B*	545	*SLC5A2*
6	*ACTB*	141	*CRTAP*	285	*HSPA9*	411	*PEX12*	546	*SLC6A19*
7	*ACTC1*	142	*CSPP1*	286	*HSPG2*	412	*PEX13*	547	*SLC6A20*
8	*ACTG2*	143	*CTC1*	287	*HYLS1*	413	*PEX14*	548	*SLC7A7*
9	*ACTN4*	144	*CTNS*	288	*IFT122*	414	*PEX16*	549	*SLC7A9*
10	*ACVRL1*	145	*CUBN*	289	*IFT140*	415	*PEX19*	550	*SLC9A3R1*
11	*ADAMTS13*	146	*CUL3*	281	*IFT172*	416	*PEX2*	551	*SLIT2*
12	*AFF4*	147	*CYP11A1*	282	*IFT27*	417	*PEX26*	552	*SLX4*
13	*AGPAT2*	148	*CYP11B1*	283	*IFT43*	418	*PEX3*	553	*SMAD3*
14	*AGT*	149	*CYP17A1*	284	*IFT80*	419	*PEX5*	554	*SMAD4*
15	*AGTR1*	150	*CYP21A2*	285	*INF2*	420	*PEX6*	555	*SMARCAL1*
16	*AGXT*	151	*CYP24A1*	286	*INPP5E*	421	*PGK1*	556	*SMARCE1*
17	*AHI1*	152	*DCDC2*	287	*INPPL1*	422	*PGM3*	557	*SMC1A*
18	*ALG8*	153	*DCHS1*	288	*INSR*	423	*PHEX*	558	*SMOC1*
19	*ALG9*	154	*DDX59*	289	*INVS*	424	*PHGDH*	559	*SNRPB*
20	*ALMS1*	155	*DGKE*	290	*IQCB1*	425	*PIEZO2*	560	*SOX11*
21	*ALPL*	156	*DHCR7*	291	*IRF6*	426	*PIGA*	561	*SOX17*
22	*AMER1*	157	*DIS3L2*	292	*ITGA3*	427	*PIGL*	562	*SOX18*
23	*AMN*	158	*DKC1*	293	*ITGA6*	428	*PIGN*	563	*SOX9*
24	*ANKS6*	159	*DLG3*	294	*ITGA8*	429	*PIGT*	564	*SPECC1L*
25	*ANLN*	160	*DLL3*	295	*ITGB4*	430	*PIK3CA*	565	*SPINT2*
26	*ANOS1*	161	*DLL4*	296	*JAG1*	431	*PIK3R2*	566	*SRCAP*
27	*AP2S1*	162	*DLX4*	297	*JAM3*	432	*PITX2*	567	*STAR*
28	*APC*	163	*DMP1*	298	*KANK1*	433	*PKD1*	568	*STK11*
29	*APC2*	164	*DNA2*	299	*KANK2*	434	*PKD2*	569	*STRA6*
30	*APOA1*	165	*DNAAF1*	300	*KANK4*	435	*PKHD1*	570	*STRADA*
31	*APOB*	166	*DNASE1L3*	301	*KANSL1*	436	*PKP2*	571	*STUB1*
32	*APRT*	167	*DNMT3B*	302	*KAT6B*	437	*PLCE1*	572	*STX16*
33	*AQP2*	168	*DPH1*	303	*KCNA1*	438	*PLG*	573	*SUCLA2*

34	*ARHGDIA*	169	*DSC2*	304	*KCNH1*	439	*PLOD1*	574	*SUGCT*
35	*ARID1A*	170	*DSG2*	305	*KCNH2*	440	*PMM2*	575	*TACO1*
36	*ARID1B*	171	*DSP*	306	*KCNJ1*	441	*PMS2*	576	*TAPT1*
37	*ARL13B*	172	*DSTYK*	307	*KCNJ10*	442	*PNPLA6*	577	*TBC1D20*
38	*ARL6*	173	*DYNC2H1*	308	*KCNJ5*	443	*POMC*	578	*TBC1D24*
39	*ARMC5*	174	*EBP*	309	*KCNQ1*	444	*POMT1*	579	*TBCE*
40	*ARNT2*	175	*EDNRA*	310	*KCTD1*	445	*POR*	580	*TBX18*
41	*ARX*	176	*EFEMP2*	311	*KDM1A*	446	*PORCN*	581	*TCTN2*
42	*ASXL1*	177	*EGF*	312	*KDM6A*	447	*PPP1R15B*	582	*TCTN3*
43	*ATP6V0A4*	178	*EHHADH*	313	*KIF14*	448	*PQBP1*	583	*TERC*
44	*ATP6V1B1*	179	*EIF2AK3*	314	*KIF7*	449	*PRKAG2*	584	*TFAP2A*
45	*ATP7A*	180	*EIF2B4*	315	*KL*	450	*PRKCD*	585	*TGFBR1*
46	*ATP7B*	181	*EMP2*	316	*KLHL3*	451	*PRODH*	586	*TGFBR2*
47	*AUH*	182	*ENG*	317	*KMT2D*	452	*PROKR2*	587	*THOC6*
48	*AVP*	183	*ENPP1*	318	*KRAS*	453	*PRPS1*	588	*TMCO1*
49	*AVPR2*	184	*EPG5*	319	*KYNU*	454	*PSAP*	589	*TMEM138*
50	*B2M*	185	*ERBB3*	320	*LAMB2*	455	*PTEN*	590	*TMEM216*
51	*B3GLCT*	186	*ERCC4*	321	*LAMB3*	456	*PTH*	591	*TMEM231*
52	*B4GAT1*	187	*ERCC6*	322	*LAMC2*	457	*PTH1R*	592	*TMEM237*
53	*B9D1*	188	*ERCC8*	323	*LCAT*	458	*PTPN11*	593	*TMEM43*
54	*B9D2*	189	*ESCO2*	324	*LDHA*	459	*PTPRO*	594	*TMEM67*
55	*BBIP1*	190	*ETFA*	325	*LDLR*	460	*PUF60*	595	*TMEM70*
56	*BBS1*	191	*ETFB*	326	*LFNG*	461	*PYGM*	596	*TNFRSF1A*
57	*BBS10*	192	*ETFDH*	327	*LMBRD1*	462	*RAB18*	597	*TNNI3*
58	*BBS12*	193	*EVC*	328	*LMNA*	463	*RAB23*	598	*TNNT2*
59	*BBS2*	194	*EVC2*	329	*LMX1B*	464	*RAB3GAP1*	599	*TNXB*
60	*BBS4*	195	*EYA1*	330	*LONP1*	465	*RAB3GAP2*	600	*TP53*
61	*BBS5*	196	*FAH*	331	*LPIN1*	466	*RAD51C*	601	*TP63*
62	*BBS7*	197	*FAM20A*	332	*LRIG2*	467	*RAI1*	602	*TPM1*
63	*BBS9*	198	*FAM20C*	333	*LRP2*	468	*RAP1A*	603	*TRAF3IP1*
64	*BCOR*	199	*FAN1*	334	*LRP4*	469	*RAP1B*	604	*TRAIP*
65	*BCS1L*	200	*FANCA*	335	*LTBP4*	470	*RB1*	605	*TRAP1*
66	*BMP4*	201	*FANCB*	336	*LYZ*	471	*RBBP8*	606	*TREX1*
67	*BMPER*	202	*FANCC*	337	*LZTFL1*	472	*RBM10*	607	*TRIM32*
68	*BMPR1A*	203	*FANCD2*	338	*MAFB*	473	*RBM8A*	608	*TRMT5*
69	*BRAF*	204	*FANCE*	339	*MAGED2*	474	*RECQL4*	609	*TRNT1*
70	*BRCA1*	205	*FANCF*	340	*MAP2K1*	475	*REN*	610	*TRPC6*

71	*BRCA2*	206	*FANCG*	341	*MAP2K2*	476	*RERE*	611	*TRPM6*
72	*BRIP1*	207	*FANCI*	342	*MAPRE2*	477	*RET*	612	*TSC1*
73	*BSCL2*	208	*FANCL*	343	*MBTPS2*	478	*RIN2*	613	*TSC2*
74	*BSND*	209	*FANCM*	344	*MEFV*	479	*RIPK4*	614	*TSR2*
75	*BUB1B*	210	*FASTKD2*	345	*MEN1*	480	*RIPPLY2*	615	*TTC21B*
76	*C1QA*	211	*FAT4*	346	*MESP2*	481	*RMND1*	616	*TTC37*
77	*C1QB*	212	*FBLN5*	347	*MIR17HG*	482	*RNU4ATAC*	617	*TTC8*
78	*C1QC*	213	*FBN1*	348	*MKKS*	483	*ROBO2*	618	*TTR*
79	*C2*	214	*FBXL4*	349	*MKS1*	484	*ROR2*	619	*TWIST2*
80	*C3*	215	*FGA*	350	*MLH1*	485	*RPGRIP1L*	620	*TXNL4A*
81	*C4A*	216	*FGF10*	351	*MMACHC*	486	*RPL11*	621	*UBE2T*
82	*CA2*	217	*FGF20*	352	*MNX1*	487	*RPL26*	622	*UBR1*
83	*CACNA1S*	218	*FGF23*	353	*MRPS22*	488	*RPL35A*	623	*UMOD*
84	*CAD*	219	*FGFR1*	354	*MSH2*	489	*RPL5*	624	*UMPS*
85	*CASP10*	220	*FGFR2*	355	*MSH6*	490	*RPS10*	625	*UPB1*
86	*CASR*	221	*FGFR3*	356	*MTM1*	491	*RPS17*	626	*UPK3A*
87	*CC2D2A*	222	*FKBP14*	357	*MUC1*	492	*RPS19*	627	*UQCC2*
88	*CCBE1*	223	*FLCN*	358	*MUTYH*	493	*RPS24*	628	*USP9X*
89	*CCDC22*	224	*FLNA*	359	*MVK*	494	*RPS26*	629	*VANGL1*
90	*CD151*	225	*FLNB*	360	*MYBPC3*	495	*RPS28*	630	*VHL*
91	*CD19*	226	*FLT4*	361	*MYCN*	496	*RPS29*	631	*VIPAS39*
92	*CD81*	227	*FN1*	362	*MYH11*	497	*RPS7*	632	*VPS33B*
93	*CD96*	228	*FOXC1*	363	*MYH7*	498	*RRM2B*	633	*WAS*
94	*CDC5L*	229	*FOXC2*	364	*MYH9*	499	*RTTN*	634	*WDPCP*
95	*CDC73*	230	*FOXF1*	365	*MYL2*	500	*RYR1*	635	*WDR19*
96	*CDKN1B*	231	*FRAS1*	366	*MYL3*	501	*RYR2*	636	*WDR34*
97	*CDKN1C*	232	*FREM1*	367	*MYO1E*	502	*SALL1*	637	*WDR35*
98	*CENPF*	233	*FREM2*	368	*NAA10*	503	*SALL4*	638	*WDR60*
99	*CEP104*	234	*FUZ*	369	*NARS2*	504	*SARS2*	639	*WDR73*
100	*CEP120*	235	*FXYD2*	370	*NBN*	505	*SBDS*	640	*WFS1*
101	*CEP164*	236	*G6PC*	371	*NECTIN1*	506	*SC5D*	641	*WNK1*
102	*CEP290*	237	*GALNT3*	372	*NEK1*	507	*SCARB2*	642	*WNK4*
103	*CEP41*	238	*GATA3*	373	*NEK8*	508	*SCN4A*	643	*WNT3*
104	*CEP83*	239	*GATA6*	374	*NEXMIF*	509	*SCN5A*	644	*WNT4*
105	*CFH*	240	*GBA*	375	*NF1*	510	*SCNN1A*	645	*WNT5A*
106	*CFHR5*	250	*GCDH*	376	*NF2*	511	*SCNN1B*	646	*WNT7A*

107	*CFI*	251	*GCM2*	377	*NHP2*	512	*SCNN1G*	647	*WT1*
108	*CHD7*	252	*GDF2*	378	*NIPBL*	513	*SCO1*	648	*XDH*
109	*CHRM3*	253	*GDNF*	379	*NLRP3*	514	*SDCCAG8*	649	*XPNPEP3*
110	*CHST14*	254	*GLA*	380	*NOTCH2*	515	*SDHAF2*	650	*XRCC4*
111	*CISD2*	255	*GLB1*	381	*NOTCH3*	516	*SDHB*	651	*XYLT2*
112	*CLCN5*	256	*GLI3*	382	*NPHP1*	517	*SDHC*	652	*YAP1*
113	*CLCNKA*	257	*GLIS2*	383	*NPHP3*	518	*SDHD*	653	*ZAP70*
114	*CLCNKB*	258	*GLIS3*	384	*NPHP4*	519	*SEMA3E*	654	*ZIC3*
115	*CLDN16*	259	*GNA11*	385	*NPHS1*	520	*SERPINH1*	655	*ZMPSTE24*
116	*CLDN19*	260	*GNAS*	386	*NPHS2*	521	*SETBP1*	656	*ZNF423*
117	*CNNM2*	261	*GNAS-AS1*	387	*NR0B1*	522	*SF3B4*	657	*ZNF687*
118	*COL18A1*	262	*GNB1*	388	*NR3C2*	523	*SHH*	658	*ADA2*
119	*COL3A1*	263	*GPC3*	389	*NSD1*	524	*SI*	659	*CCNQ*
120	*COL4A1*	264	*GRHPR*	390	*NSDHL*	525	*SIX1*	660	*CILK1*
121	*COL4A3*	265	*GRIP1*	391	*NUP107*	526	*SIX2*	661	*COA8*
122	*COL4A4*	266	*GSN*	392	*NUP205*	527	*SIX5*	662	*CPLANE1*
123	*COL4A5*	267	*HBB*	393	*NUP93*	528	*SLC12A1*	663	*ELP1*
124	*COL5A1*	268	*HDAC8*	394	*OCLN*	529	*SLC12A3*	664	*LARS1*
125	*COPA*	269	*HES7*	395	*OCRL*	530	*SLC16A12*	665	*MMUT*
126	*COQ2*	270	*HGD*	396	*OFD1*	531	*SLC1A1*		
127	*COQ6*	271	*HNF1A*	397	*OPLAH*	532	*SLC22A12*		
128	*COQ7*	272	*HNF1B*	398	*OTC*	533	*SLC25A1*		
129	*COQ8B*	273	*HNF4A*	399	*PAF1*	534	*SLC26A4*		
130	*COQ9*	274	*HOGA1*	400	*PALB2*	535	*SLC2A10*		
131	*COX10*	275	*HOXA13*	401	*PAX2*	536	*SLC2A2*		
132	*COX14*	276	*HOXD13*	402	*PC*	537	*SLC2A9*		
133	*COX20*	277	*HPRT1*	403	*PCSK9*	538	*SLC34A1*		
134	*COX6B1*	278	*HPS1*	404	*PDE6D*	539	*SLC34A3*		
135	*COX7B*	279	*HPSE2*	405	*PDSS1*	540	*SLC36A2*		

Supplementary Table S2: Genes analyzed by whole exome sequencing.

**Antibody**	**Host species**	**Dilution**	**Company/Catalog Number**
Anti-CLC-K	Rabbit	1:200	Alomone Labs (#ACL-004)
Anti-Barttin	Rabbit	1:500	Gift
Anti-NKCC2	Guinea pig	1:500	Schmitt, et al. (1)
Anti-NCC	Rabbit	1:500	Gift
Anti-total SPAK	Rabbit	1:100	Cell Signaling (#2281)
Anti-Parvalbumin	Rabbit	1:1000	ThermoScientific (#PA1-933)
Anti-Calbindin	Rabbit	1:2000	Chemicon International (#AB1778)
Anti-HA High Affinity	Rat	1:5000	Roche #3F10
Anti-α-Tubulin	Mouse	1:5000	Sigma-Aldrich #T6074
Anti-Cadherin	Rabbit	1:1000	Sigma-Aldrich #C3678
Anti-GAPDH	Rabbit	1:1000	Cell Signaling #5174

Supplementary Table S3. Characteristics of primary antibodies used in this study.

Age (years)	Sex (M/F)	Race/ Ethnicity	Diagnosis	Serum Creatinine (mg/dL)	Serum K^+ (meq/L)
19	M	Hispanic	Gunshot wound	1.2	4.7
24	M	Black	Gunshot wound	1.2	3.3
16	M	Filipino	Refractory hypokalemia, chronic kidney disease	1.1-1.3	1.7
17 (explant)	M	Filipino	Refractory hypokalemia, chronic kidney disease	2.57	2.2

Supplementary Table S4: Clinical characteristics of controls vs. samples from index patient (shaded) at time of sample collection.

Sample	Fractional volume of distal convoluted tubule (%)
Index- Nephrectomy	5
Index- Explant	5.7
Control- 1	11.7
Control- 2	15.8

Supplementary Table S5: Comparison of distal convoluted tubule from control vs. samples from index patient.

SUPPLEMENTARY METHODS

Structure Analysis and Data availability

We used Pymol (Schrödinger) to describe the structure of CLC-K (PDB:5TR1). The PDB file is available at www.rcsb.org. The distance between Gly-167 and Cl-binding site was calculated using Pymol.

SUPPLEMENTARY REFERENCES

1. Schmitt R, Klussmann E, Kahl T, Ellison DH, Bachmann S. Renal expression of sodium transporters and aquaporin-2 in hypothyroid rats. *Am J Physiol Renal Physiol*. 2003;284(5):F1097-1104. doi:10.1152/ajprenal.00368.2002
