# Supplementary material for: ClC-Kb pore mutation disrupts glycosylation and triggers distal tubular remodeling
Source: JCI Insight. 2024 Nov 22;9(22):e175998. doi: 10.1172/jci.insight.175998 (PMC11601903; doi:10.1172/jci.insight.175998)
Supplement: Unedited blot and gel images [file jciinsight-9-175998-s108.pdf]

Full blot for Figure 3A

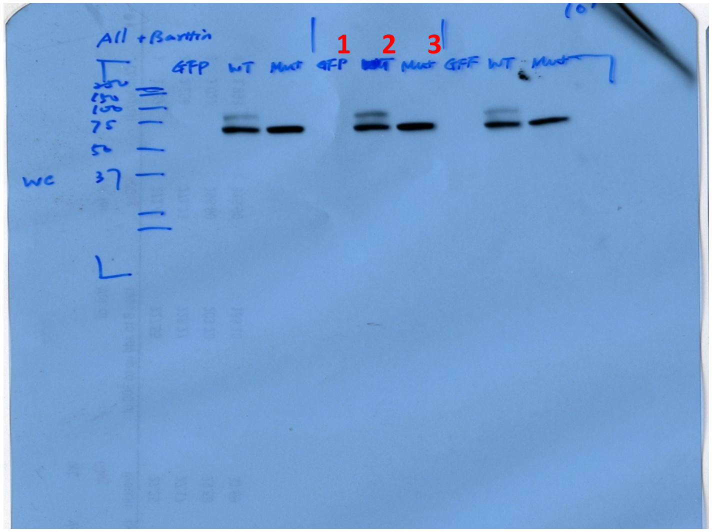

Anti-HA

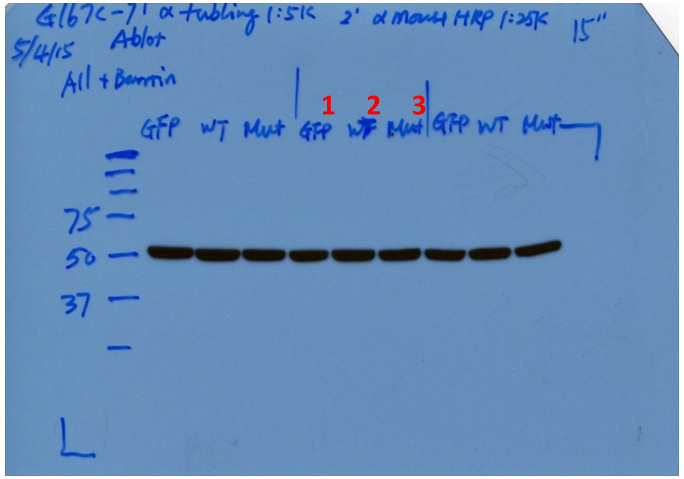

anti-α-tubulin

Full blot for Figure 3C

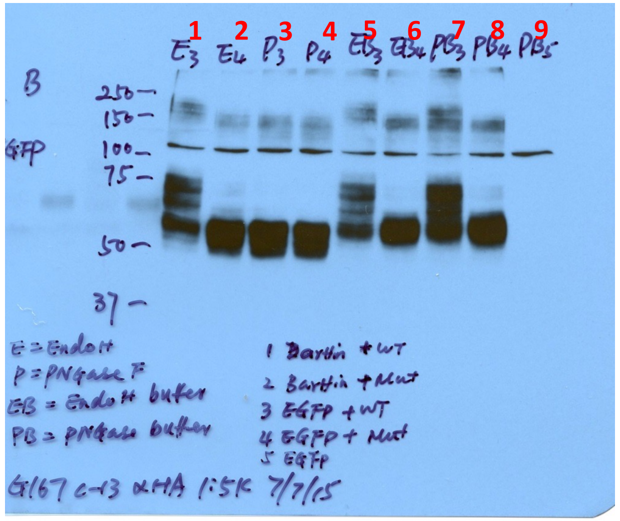

Anti-HA

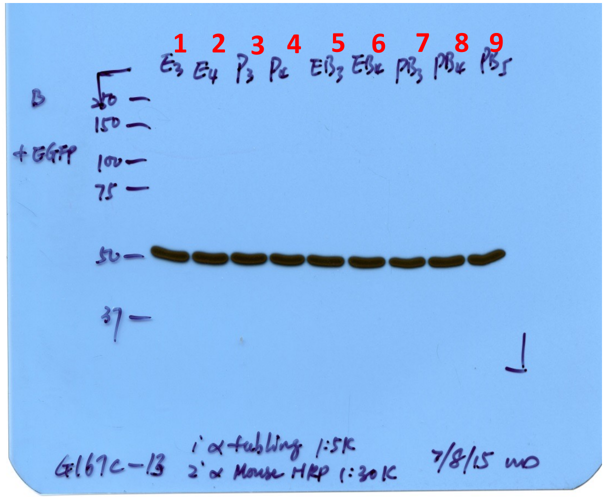

anti-α-tubulin

Full blot for Figure 4E

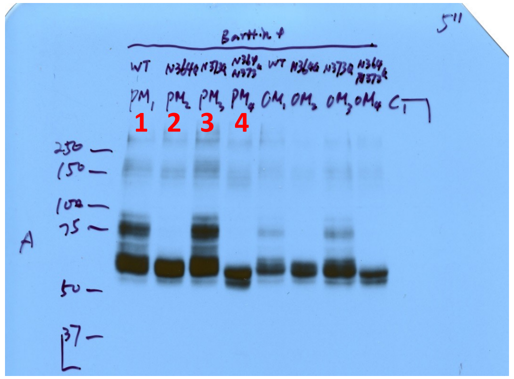

Anti-HA

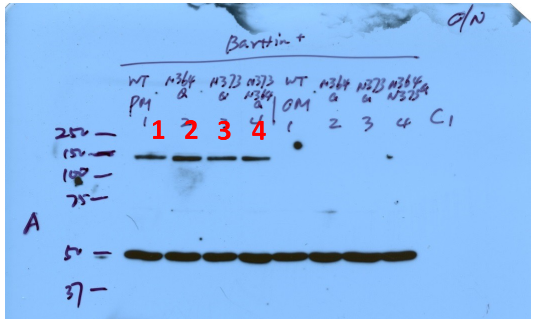

anti-cadherin

Full blot for Figure 5A

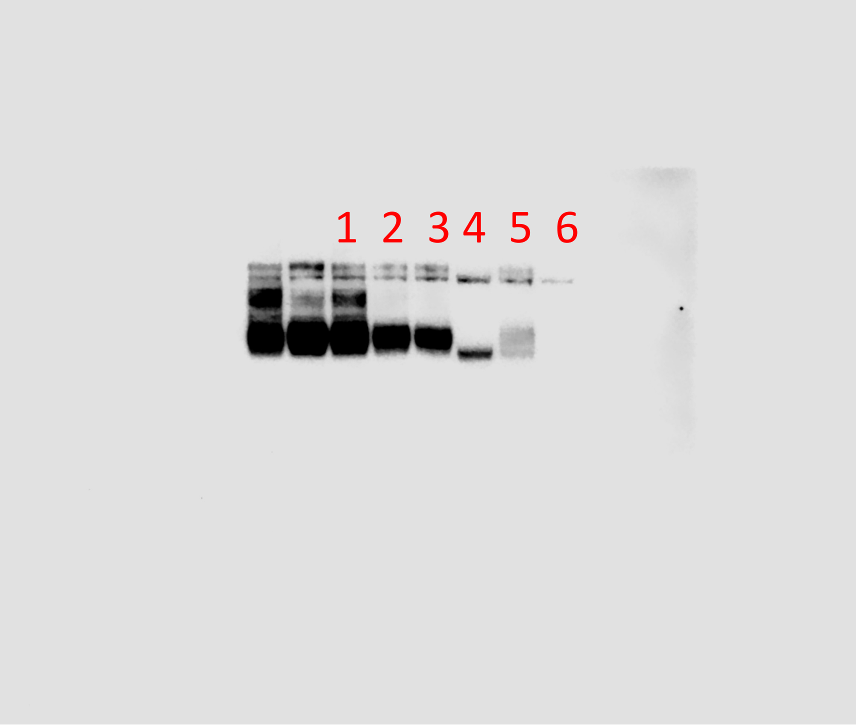

Anti-HA

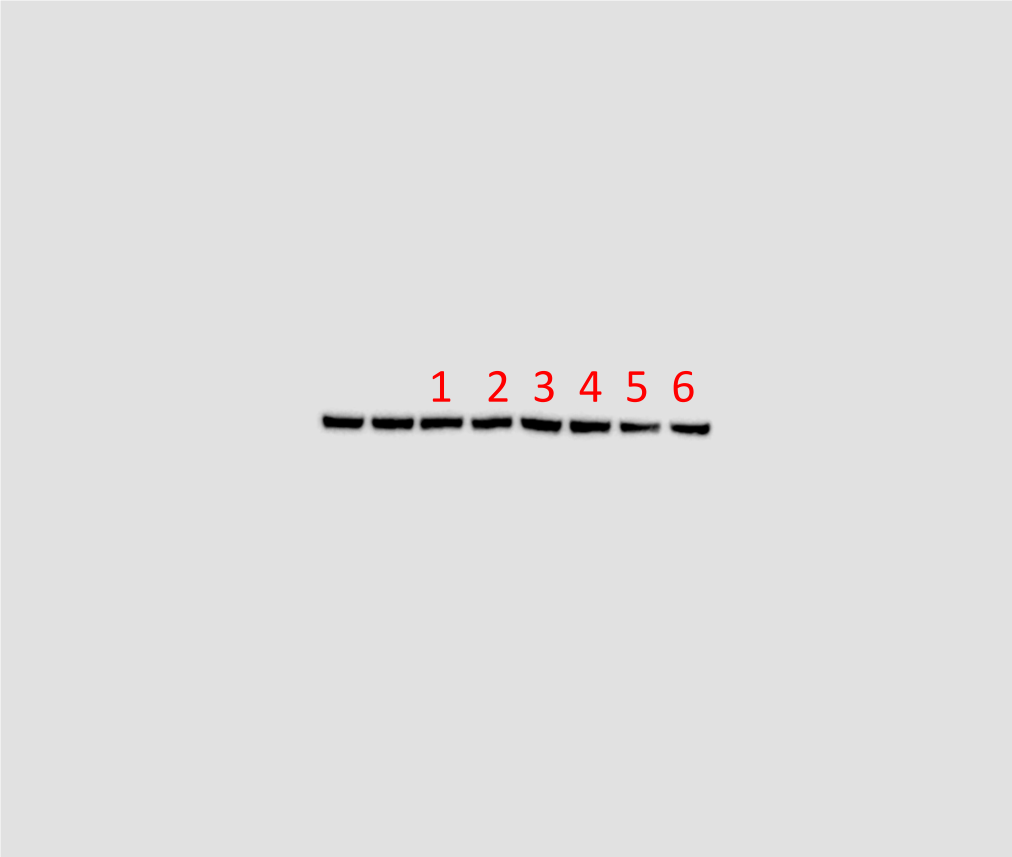

Anti-tubulin

Full blot for Figure 5C

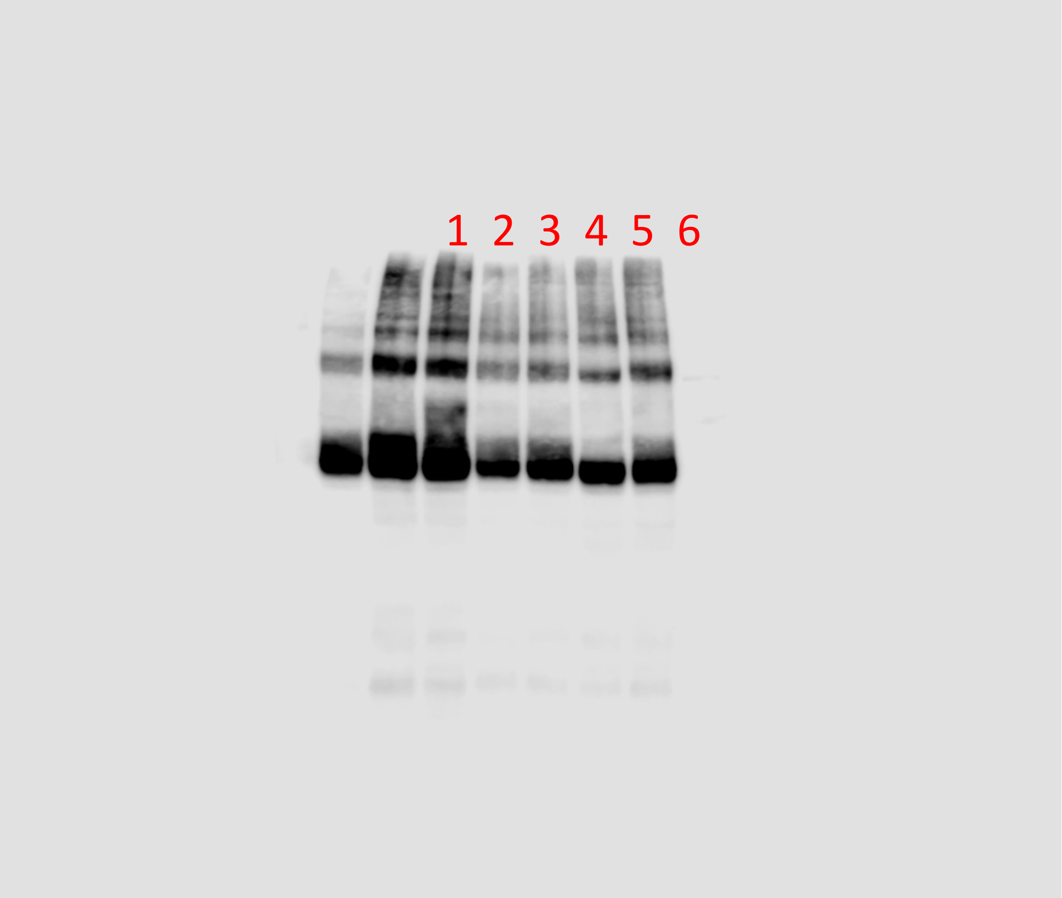

Anti-HA

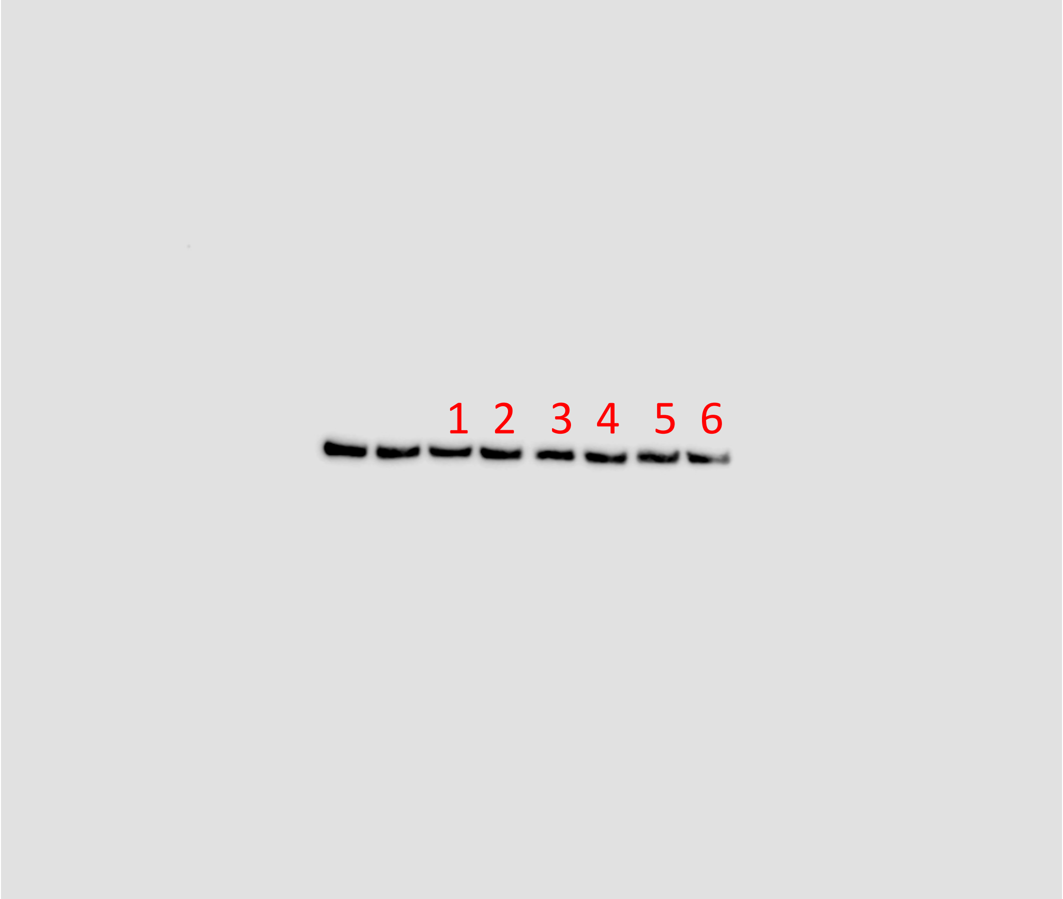

Anti-GAPDH

Full blot for Figure 6A

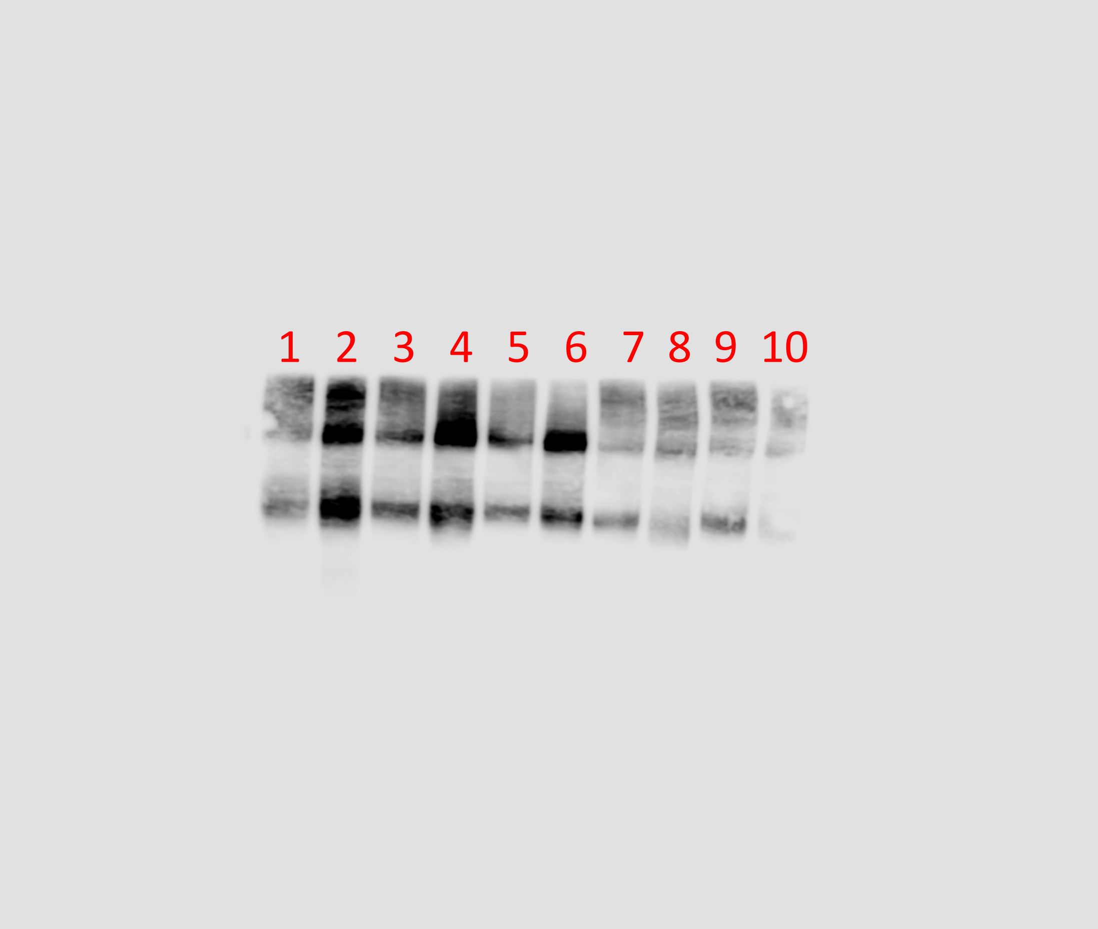

Anti-HA

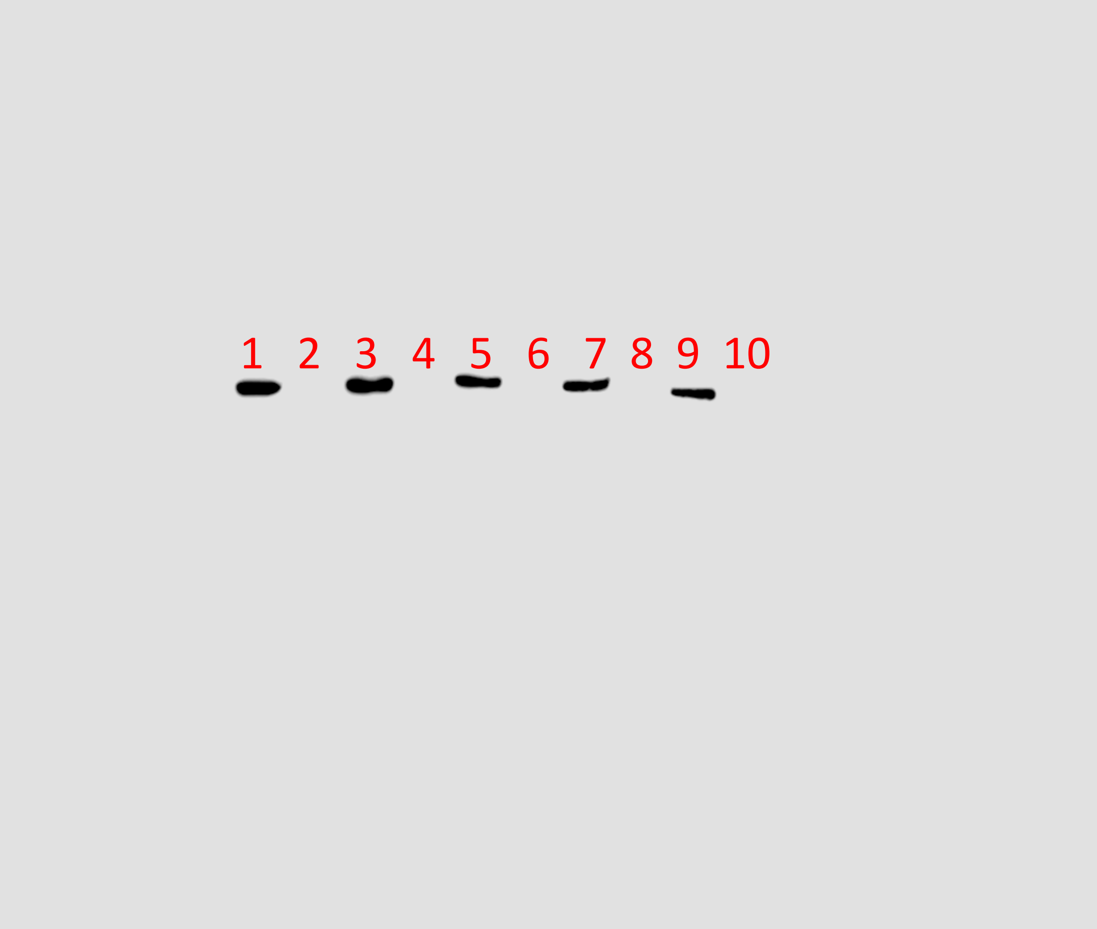

Anti-GAPDH

Full blot for Figure 6C

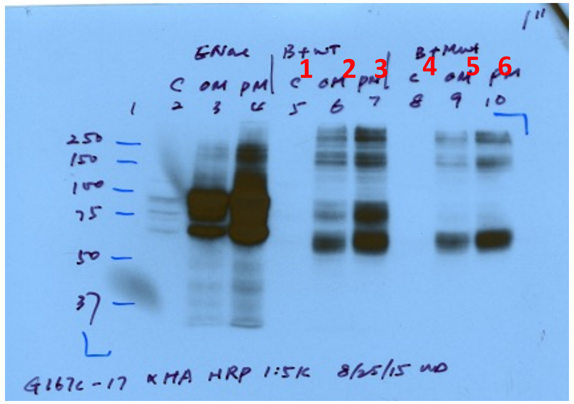

Anti-HA

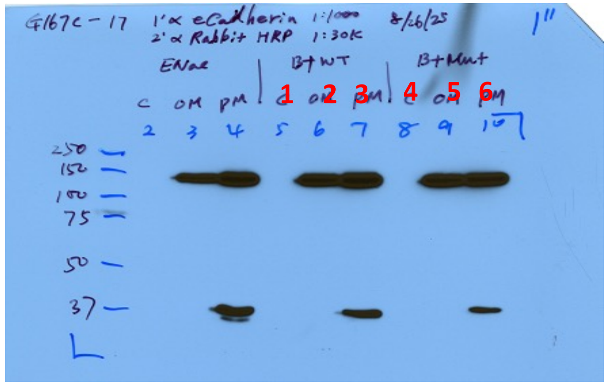

anti-cadherin
